# Development of a Risk Characterization Tool for Harmful Cyanobacteria Blooms on the Ohio River

**DOI:** 10.3390/w14040644

**Published:** 2022-02-18

**Authors:** Christopher T. Nietch, Leslie Gains-Germain, James Lazorchak, Scott P. Keely, Gregory Youngstrom, Emilee M. Urichich, Brian Astifan, Abram DaSilva, Heather Mayfield

**Affiliations:** 1 USEPA Office of Research and Development, Center for Environmental Measurement and Modeling, 26 Martin Luther King Dr W, Cincinnati, OH 45268, USA; 2 Neptune and Company, Inc., 1435 Garrison Street, Suite 201, Lakewood, CO 80215, USA; 3 Ohio River Valley Water Sanitation Commission, 5735 Kellogg Ave., Cincinnati, OH 45230, USA; 4 National Weather Service, Ohio River Forecast Center, 1901 South State Route 134, Wilmington, OH 45177, USA; 5 Foundation for Ohio River Education, Ohio River Valley Water Sanitation Commission, 5735 Kellogg Ave., Cincinnati, OH 45230, USA

**Keywords:** cyanobacteria, big river, harmful algae bloom, risk characterization, predictive modeling

## Abstract

A data-driven approach to characterizing the risk of cyanobacteria-based harmful algal blooms (cyanoHABs) was undertaken for the Ohio River. Twenty-five years of river discharge data were used to develop Bayesian regression models that are currently applicable to 20 sites spread-out along the entire 1579 km of the river’s length. Two site-level prediction models were developed based on the antecedent flow conditions of the two blooms that occurred on the river in 2015 and 2019: one predicts if the current year will have a bloom (the occurrence model), and another predicts bloom persistence (the persistence model). Predictors for both models were based on time-lagged average flow exceedances and a site’s characteristic residence time under low flow conditions. Model results are presented in terms of probabilities of occurrence or persistence with uncertainty. Although the occurrence of the 2019 bloom was well predicted with the modeling approach, the limited number of events constrained formal model validation. However, as a measure of performance, leave-one-out cross validation returned low misclassification rates, suggesting that future years with flow time series like the previous bloom years will be correctly predicted and characterized for persistence potential. The prediction probabilities are served in real time as a component of a risk characterization tool/web application. In addition to presenting the model’s results, the tool was designed with visualization options for studying water quality trends among eight river sites currently collecting data that could be associated with or indicative of bloom conditions. The tool is made accessible to river water quality professionals to support risk communication to stakeholders, as well as serving as a real-time water data monitoring utility.

## 1. Introduction

The intensity, frequency, and duration of harmful cyanobacteria blooms (cyanoHABs) appear to be increasing in freshwater habitats across the globe [1,2]. Most attention has been focused on blooms in lakes and reservoirs, including the Laurentian Great Lakes [3]. CyanoHABs result in significant socio-economic impact [4] and can pose significant risk to the safety of drinking water and public health from direct contact with people and their pets [5,6]. They can cause taste and odor problems for drinking water treatment [7], but their harmful nature is largely attributed to the toxins that many species of the cyanobacteria are capable of producing, including neurotoxins, hepatotoxins, and dermatoxins [8].

The expanding awareness, if not actual number, of freshwater cyanoHABs reported over the last couple of decades has stimulated an intense amount of research to better understand their ecology for management and prevention [9,10]. This research effort has solidified the connection between the rise in cyanoHABs in freshwaters and excess loading of nitrogen and phosphorus [11–15] (hereafter referred to collectively as nutrients), as well as the changing climate [16]. The former has accompanied the cumulative effects of intense agricultural activity and increases in runoff from urban areas [9,17–20], while the latter is contributing to the warming of surface waters and changing precipitation [3,21–25]. The changing climate is producing more optimal growth conditions for cyanobacteria compared to other phytoplankton species [26,27] and is affecting important watershed loading and lake biogeochemical processes that control nutrient availability [13,28–33].

CyanoHABs appear to be impacting lentic systems the most, but lotic systems are also susceptible [34,35]. Large regulated rivers are vulnerable to cyanobacteria blooms, where they are found to concentrate behind flow control structures [36]. However, less is understood about cyanobacteria and toxin dynamics in lotic systems [37,38]. Because of the relatively higher hydraulic flushing rates of river environments, the susceptibility of most of these systems to cyanoHAB events has only recently become a concern [39,40].

An unprecedented bloom event on the Ohio River in 2015 brought the potential risks of cyanoHABs in larger rivers of the United States into the spotlight [41–43]. It was first observed in the upper river on 19 August, 135 km from the river’s origin at the Pike Island Lock and Dam (L&D), just upstream of Wheeling, WV. The bloom reports increased in the subsequent days in a downstream direction. Thirty days later, it was first observed near the river’s confluence with the Mississippi River at Paducah, KY. By the end of September, water recreation advisories or precautionary statements had been posted for 1127 km of the 1579 km-long river by the States of Illinois, Indiana, Kentucky, Ohio, and West Virginia. The last advisory was not lifted until 3 November.

There are 32 municipal drinking water treatment plants (DWTPs) on the Ohio River, serving 5 million people. Over the course of the 2015 cyanoHAB, 20 of the DWTPs had to take precautions by providing additional treatment, increasing monitoring, or closing their intakes. The extra treatment cost was estimated at $2 million USD based on a survey of utilities conducted by staff of the Ohio River Valley Water Sanitation Commission (ORSANCO), which administers the provisions of an interstate compact that includes a responsibility of monitoring water quality in the Ohio River. The Greater Cincinnati Water Works, alone, reported additional treatment costs of 7000.00 USD per day for two months. The 2015 cyanoHAB caught water resource professionals and drinking water utility managers off-guard, creating much interest in understanding the cause of the event and in implementing risk management procedures and practices to be better prepared for subsequent ones. Risk management and mitigation approaches used for larger lakes may not easily translate to flowing water systems. For instance, the long and narrow configurations of river systems limit the use of satellite-based risk characterization at present [44–47]. This means alternative approaches are necessary for managing the risk posed by cyanoHABs in flowing waters.

This research was initiated in response to this HABs risk management need. The study goal was to produce a tool that could be used by ORSANCO for assessing the potential for cyanoHABs in real time and at multiple locations along the river’s length. When the project started, the effort was constrained by the fact that there was only one cyanoHAB event ever documented on the Ohio River. However, in 2019, the river experienced another bloom. This event was smaller (ca. 482 km) and did not last quite as long: it was first reported on 11 September and lasted about 30 days. Prior to the 2015 and 2019 events, the largest algal bloom on the Ohio River was believed to have occurred in 2008, covering 48 km and lasting about 10 days. However, this event could not be verified as a cyanoHAB based on the available grab sampling data [41]. We hypothesized that we could take advantage of the physiography of the Ohio River along with its dense hydrologic sensing network to develop a risk characterization tool applicable to the entire river in support of ORSANCO’s cyanoHABs risk management goals. The central science question was how to best model the risk given the available data and the urgent need.

## 2. Materials and Methods

### 2.1. Ohio River State and Water Data Sources

The Ohio River receives direct discharges of nutrients from 182 wastewater treatment plants that are permitted for a combined daily load of nearly 1 billion gallons, although typically individual plants operate lower than their permitted level. There are forty-nine communities along the river with combined sewers, which may release untreated wastewater during large rainfall/runoff events through 965 outfalls. Additionally, there are 69 communities with municipal separate storm sewer systems draining urban runoff to the river. Furthermore, nutrients enter the river from the roughly 40% agricultural land cover of the 528,204 km^2^ watershed. The river is also strongly impacted by industrial discharges [48].

The model development component of the risk characterization tool needed to be tied to current and actively maintained water data acquisition. However, it would have to depend on historic data of the same type as that currently acquired, as both current and older data would be used to test assumptions and hypotheses about the controlling factors and ecological responses related to the river’s major cyanoHAB events. Several entities have water monitoring stations on the mainstem of the Ohio River. Of these, we focused attention on sources that were likely to have consistent, long-term, and current information available. These included the U.S. Geological Survey (USGS), the U.S. Army Corps of Engineers (USACE), The Ohio River Forecast Center (OHRFC), ORSANCO, and several drinking water treatment utilities. Supplemental text offers an overview of the data from each of these sources. In brief, while we found data from ORSANCO’s routine nutrient grab sampling and pool volume estimates provided by USACE useful for model development, only the data on river flows met both the spatial and temporal criteria of appropriateness for model development. No water quality (WQ) and temperature data met the criteria for modeling, but a framework, subsequently described, was developed for incorporating these data as diagnostics into the risk characterization tool.

### 2.2. Compiling River Discharge Time Series

**Figure 1.**
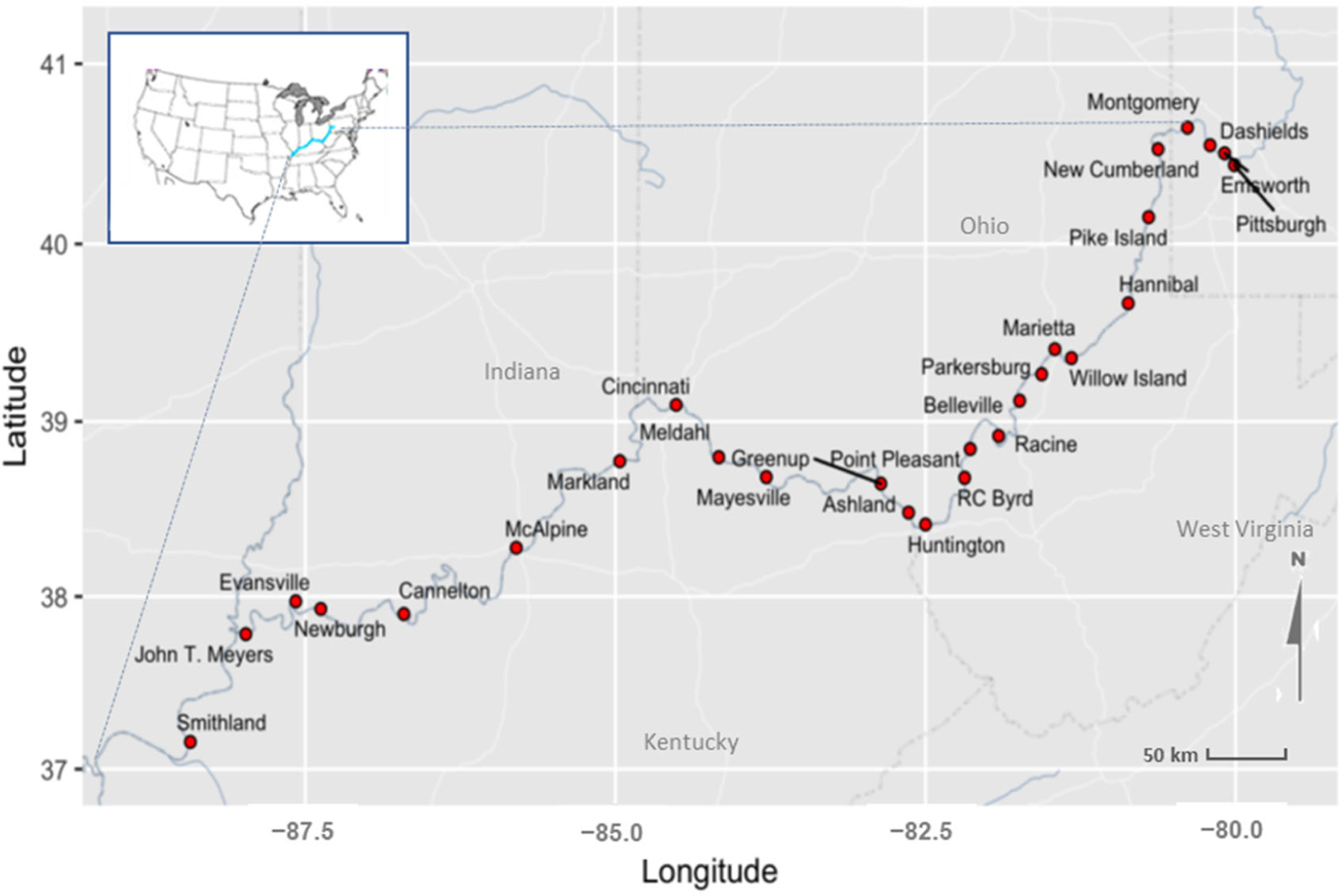
Site locations along the Ohio River where historical and real-time flow data were evaluated for modeling.

Starting with 25 years of river stage data, we focused our attention on identifying locations where discharge estimates over the whole range of stages were likely to be the most accurate. There were 18 L&D locations that met the data requirements (Figure S1). Each one had stage data for the upstream-pool and tailwater of the dams. Additionally, nine non-L&D, mid-pool stage gauging stations were evaluated (Table 1, Figure 1). For all river stage gauges, we verified that using stage as a proxy for flow would provide reasonable estimates over the range of low and high river flows and be minimally impacted by backwater and tributary influences (see supplemental text and Figure S2). This discharge estimation filter, applied at each of the 27 sites with stage data, disqualified upper pool stations and eliminated one L&D and mid-pool site entirely (J.T. Meyers and Pittsburgh, respectively). Discharge estimates from five other mid-pool gauges were considered less accurate in the lower flow ranges. These were useful for model development because their inclusion made it so there was not complete separation (i.e., a bloom occurs under different flow conditions). However, we do not include these five sites in the real-time reporting of model predictions (Table 1).

### 2.3 Guiding Conceptual Model of cyanoHAB Ecology

**Figure 2.**
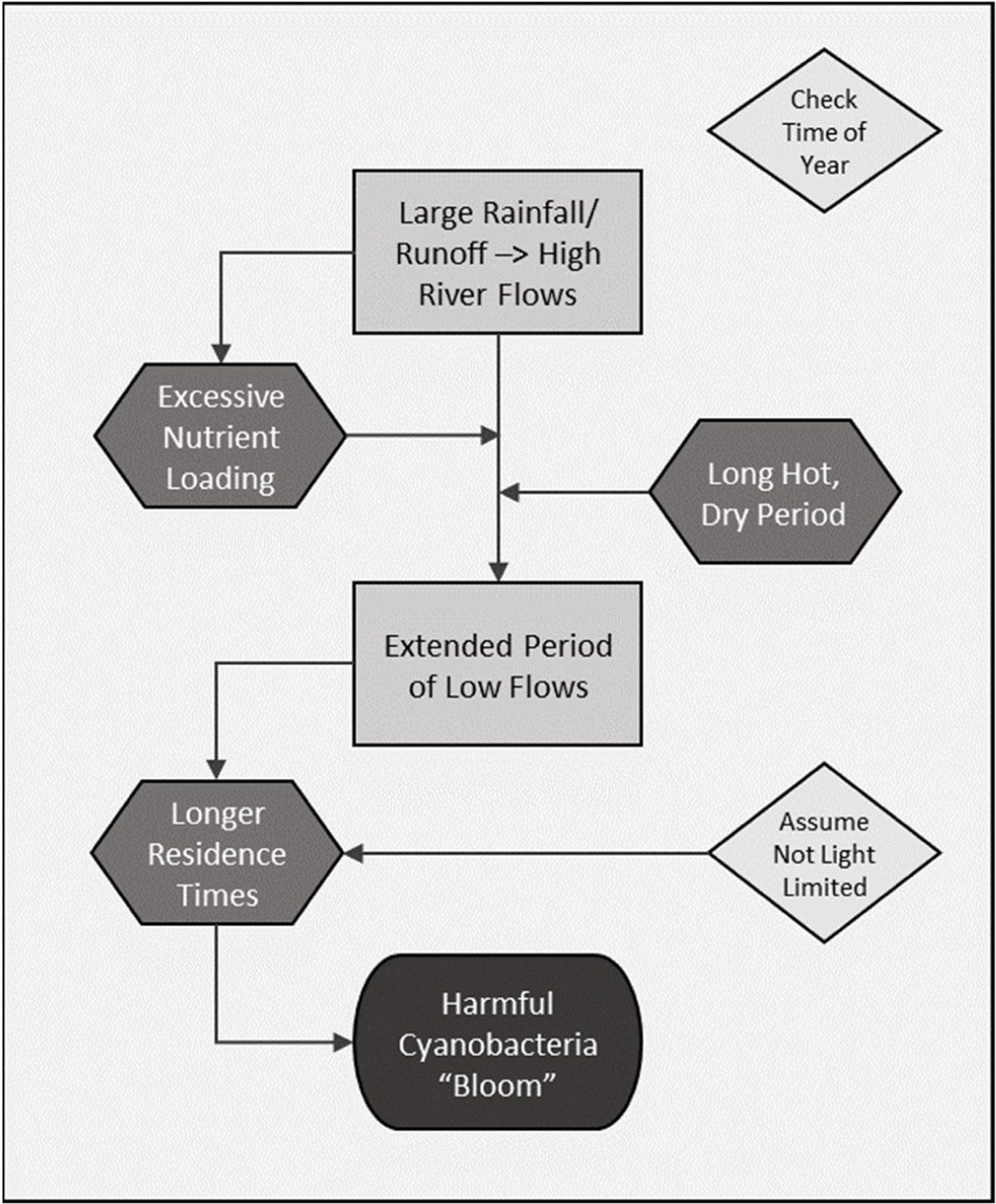
Conceptual cause and effects model linking cyanoHAB topreceding river flow conditions.

**Figure 3.**
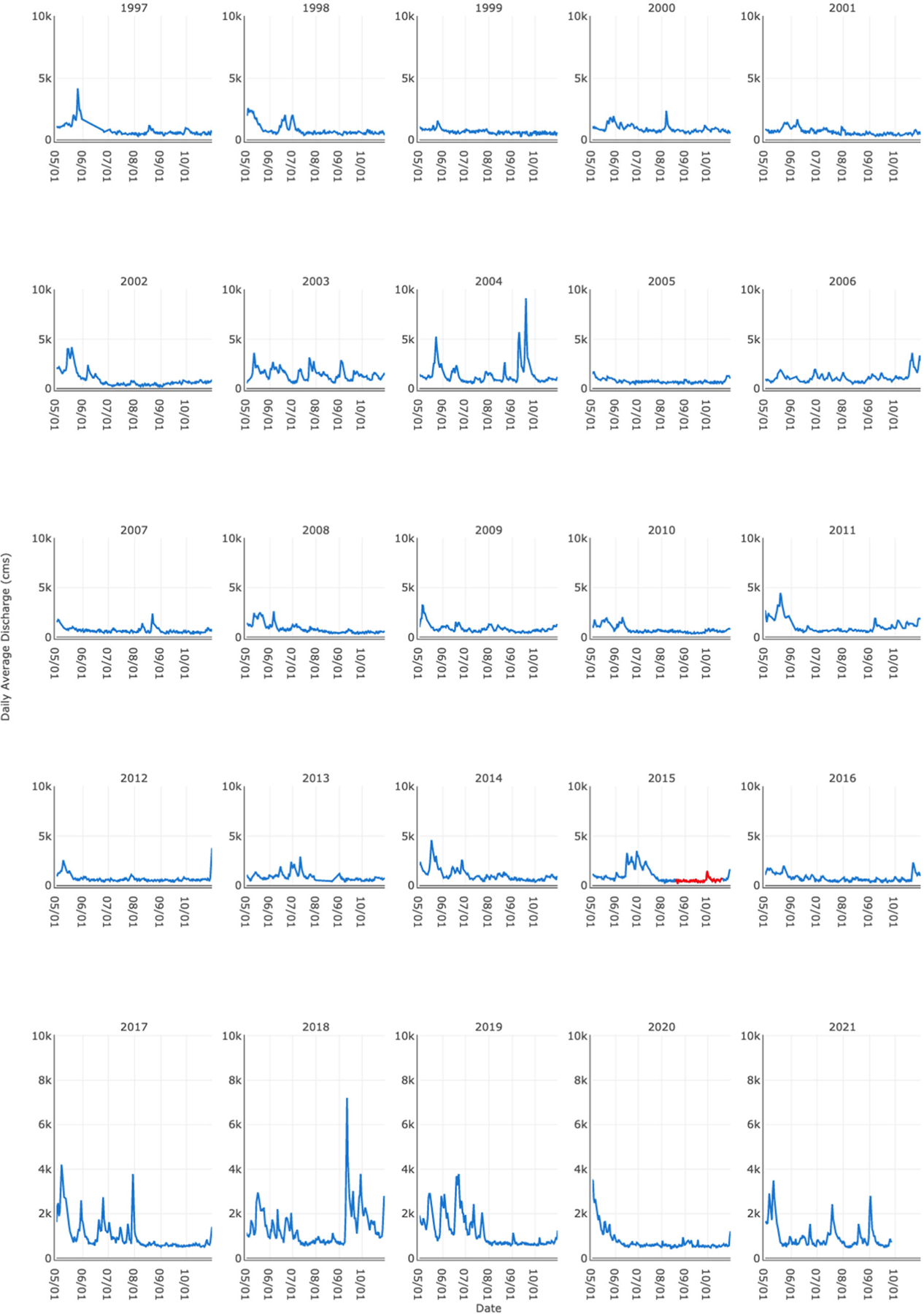
Example visualization approach to identify uniqueness of flow conditions during bloom years. Average daily discharge data for the Pike Island site plotted for 1995 through 2021, beginning of May to the end of October each year. Bloom first reported at Pike Island in 2015 (points in red signify bloom period).

With the time series of discharges, we wanted to determine if the 2015 cyanoHAB could be related to unique flow conditions, which, in turn, could be used as a basis for risk modeling. We produced a conceptual cause and effect model to guide the analysis of the empirical data (Figure 2). The conceptual model postulated that persistent low flow conditions following a period of relatively high flows are required for cyanoHAB development. The reasoning was that high flows deliver nutrients to the river that are needed to fuel cyanobacteria growth. After flows have decreased significantly for an extended period, this allows the increased growth to concentrate before being flushed down river. Thus, we propose that an increase in water residence time in the pools behind the L&Ds under presumably high nutrient availability is key to CyanoHAB formation. This logic derives from the 2015 summer flow dynamics, during which a relatively late period of high flows that occurred in June and early July was followed immediately by a period of low flows under hot and dry mid and late summer ambient conditions. The preceding high flow period was hypothesized as critical because there were several instances in previous years during which an extended period of low flows occurred, and presumably at water temperatures conducive to cyanobacteria proliferation, yet no blooms were reported (Figure 3). We assume seasonal temperature patterns and water clarity act as constraints on bloom development, with bloom potential only realistic in the summer months and when turbidity does not limit light availability for phytoplankton photosynthesis.

### 2.4 Model Development

First, we had to decide how a bloom would be defined for modeling. When the effort began, we had either zero or one bloom occurrence at each site, as only the 2015 event had taken place at this time. Defining the precise beginning and end of a bloom anywhere on the river is uncertain in practice: Blooms thus far have been first reported by river observers on seeing atypically green waters or surface scums. Once reported, the suspected bloom is subsequently sampled to verify if toxin-producing cyanobacteria are present and to test for toxins. If toxins are detected, then “bloom condition” status remains in effect from a risk management perspective until two consecutive samples collected a week apart are toxin-free. This means that, in practice, bloom status is operationally disconnected from actual dynamics of cyanobacteria cell densities. Therefore, we had no choice other than to model a binary response (i.e., 1 = bloom, 0 = no bloom), one based on when an observational report of a bloom was first received on the front end and the date when river sections were considered toxin-free on the back end (Table 1). Therefore, bloom occurrence is defined as the report of a bloom event that has been verified as toxic. Each day in a site’s time series of flows is categorized with 1 or 0, with 1 signifying the presence of a bloom. Because of the lag between the day a bloom was first reported and when the bloom was verified as toxic, we assumed the bloom was toxic on the day of the report, classifying the day the bloom report was received with 1. The site’s bloom status remains as 1 until no toxin is detected. Because of the limited number of bloom events, any given site’s time series of flows is mostly classified with zeros.

Other key modeling considerations including both assumptions and data characteristics were the availability of 25 years of flow data at 25 locations on the river; spatial and temporal correlations; that there was no “true” replication; and an assumption that years are independent. We desired a model that would estimate the probability that the current year at a given site would be a bloom year, and that could be quantified as such on any given day during the period that a cyanoHAB might occur on the river, which we qualified as May through October. We acknowledge that because blooms have occurred so infrequently on the Ohio River (i.e., only in 2015 and 2019), this constrains the reliability of the model for future occurrences. With this acknowledgment, our approach to model development proceeded with the notion that the modeling component of the risk characterization tool would be updated annually as more blooms occur in the future.

#### 2.4.1 Predictor Variables

To normalize flow rates across sites, we used “exceedances”, which are rankings based on the percentage of maximum flow for the period of record. The lowest flow at a site has an exceedance of 100%, and the highest flow has an exceedance of 0%, so high flows are associated with low exceedances, and low flows are associated with high exceedances. During an exploratory phase, we developed temporal lag terms using the exceedances to reflect the hypothesized flow conditions required for a cyanoHAB. The first lag term was meant to capture the period of low flows preceding a bloom. We started with averaging the previous 30 days of daily average flow exceedances at a site for all days in the site’s time series. When average flow rates are very low over this lag period, the exceedances are high, and, therefore, the value of this term is high. A second lag term was developed to reflect the period of high flows prior to the 30 day low flow period. To start, we calculated an average exceedance based on a 30 to 75 day lag period. The lower this average exceedance, the higher the average flows would have been for this period 30 days prior to the bloom. We then took a ratio of these two lag terms to characterize the hypothesized prerequisite conditions as one variable. A relatively high value of the ratio of the early lag term over the later one indicates a period preceding any given point in the time series when very low flows were preceded by very high flows.

Plotting the ratio of lagged exceedance terms suggested that the ratio entered a rapidly increasing phase that appeared unique for the 2015 bloom year. We hypothesized that producing another predictor variable that quantified the extent the ratio was increasing prior to a bloom event would likely improve model fit.

In the preliminary model fitting phase, we used a mixed effects binary logistic regression approach for rapid testing. Because the averaging periods of the exploratory phase’s lag terms were arbitrary based on visualizing the discharge time series (Figure 3), we systematically tested the fit of different averaging windows of time for the combination of the two lag terms to determine which would likely provide the best fit. We also tested different expressions for the period prior to the bloom when the ratio of lagged exceedances was increasing. A relative rate of increase was computed using linear regression and was tested, as was a simple count of the number of days the ratio was increasing in the prior 15 days. The Akaike Information Criterion (AIC) from a mixed effects model with the same predictors as above was used to rate the performance of different lag term windows, which differed from 1 to 15 through 25 days for the proximal period and changed from 15 through 25 to 50 through 60 days prior, with 1 to 5 day gaps considered between the two windows. The mixed effects model was fit using R 3.5.1 and the glmer function within the lme4 package v1.1 [49].

**Figure 4.**
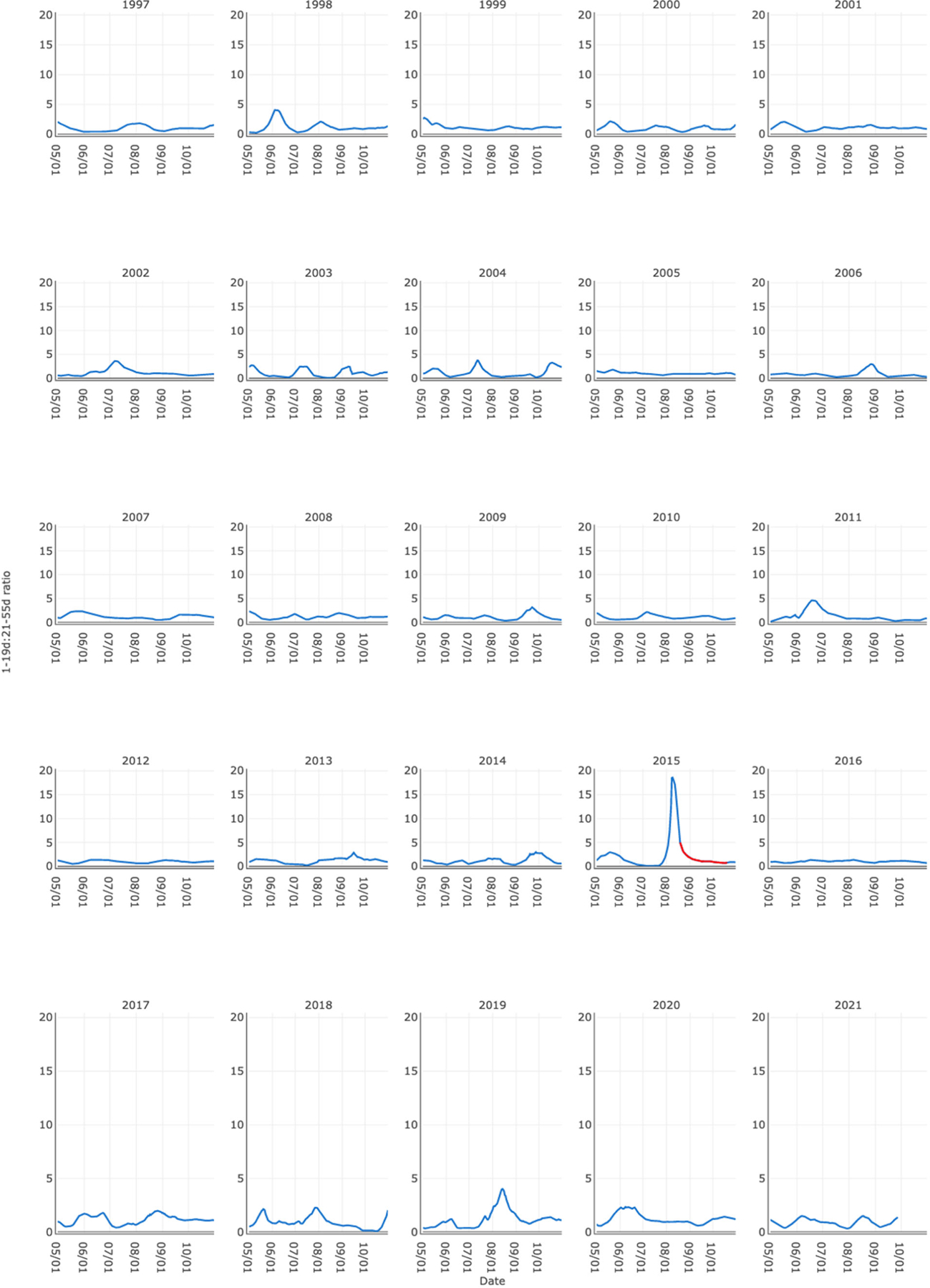
Yearly plots of the ratio of the 1 to 19 day and 21 to 55 day average lagged exceedances at Pike Island.

Using the slope rather than a simple count to characterize the ratio’s increasing phase did not improve the model fit, and the 1 to 19 day and 21 to 55 day ratio significantly outperformed all other lag term combinations (i.e., AIC lower by 2.0 compared to other combinations). Figure 4 plots the ratio of the best fit lag terms at the Pike Island site as an example. The uniqueness of 2015 is a standout. We refer to the predictor variable “*maxratio*” as the maximum 1–19:21–55 ratio that occurred prior to the 2015 cyanoHAB start date at a site. For other years, the *maxratio* is defined as the maximum ratio that occurred at any time during which a cyanoHAB is deemed possible (i.e., May through October). We defined the predictor variable “*inc15*” as the number of days in the 15 days prior to the day the *maxratio* occurred over which the ratio increased. The predictors are summarized yearly, but probabilities are estimated daily by using daily 1–19:21–55 day ratio and number of increasing ratio days.

We also considered an estimate of a site’s residence time as a potentially important predictor. This was based on our conceptual model (Figure 2) and the observation that sites upstream of Pike Island, where the bloom was first reported, were not affected by the 2015 cyanoHAB (Table 1). We hypothesized that residence times at these upriver sites were significantly shorter than those in the 19 days prior to when the bloom was reported at Pike Island and compared to other sites downstream. Therefore, when a site’s residence time is low despite a low flow condition, we’d be less likely to expect a bloom. Residence times were computed for each site by estimating the pool volume upstream of the site and the next lock and dam. A site’s upstream characteristic pool volume was provided by partners from USACE (Erich Emery and Robert Boyer, pers. comm.). Dividing a pool volume by average daily discharge resulted in a daily residence time. The variable “*meanrt*” was calculated as the mean residence time during the first (1–19 day) lag period in 2015. It is an integer between 0 and 15 and it does not change year to year.

### 2.4.2. Occurrence Model

We used Bayesian hierarchical regression for modeling. With Bayesian inference, the credible interval defines bloom prediction probability. The hierarchy is defined by year and site-specific predictors. The occurrence model responses are yearly summaries of whether a bloom occurred or not at each location for the years 1995 to 2020. Responses are assumed to follow a Bernoulli distribution, and the model is defined with a logit link function. The logit is the logarithm of the odds of bloom occurrence where odds are defined by the probability of bloom occurrence divided by the probability of bloom absence $\left(\frac{\text{p}}{1-\text{p}}\right)$.

(1) $$\text{logit}\left({\text{p}}_{\text{si}}\right)={\text{α}}_{0\text{s}}+{\text{β}}_{0}+\left({\text{α}}_{1\text{s}}+{\text{β}}_{1}\right){\text{X}}_{1}+{\text{β}}_{2}{\text{X}}_{2}+{\text{β}}_{3}{\text{X}}_{3}+{\text{β}}_{4}{\text{X}}_{4}\;{\text{α}}_{\text{0s}}\text{\textasciitilde{} Normal }\left({\text{0, σ}}_{\text{0}}\right)\;{\text{α}}_{\text{1s}}\text{\textasciitilde{} Normal }\left({\text{0, σ}}_{\text{1}}\right)\;{\text{β}}_{\text{0}}\text{\textasciitilde{}}\;\text{Cauchy}\left(\text{0, 2.5}\right)\;{\text{β}}_{\text{1}}\text{\textasciitilde{}}\;\text{Cauchy}\left(\text{0, 2.5}\right)\;{\text{β}}_{\text{2}}\text{\textasciitilde{}}\;\text{Cauchy}\left(\text{0, 2.5}\right)\;{\text{β}}_{\text{3}}\text{\textasciitilde{}}\;\text{Cauchy}\left(\text{0, 2.5}\right)\;{\text{β}}_{\text{4}}\text{\textasciitilde{}}\;\text{Cauchy}\left(\text{0, 2.5}\right)\;{\text{σ}}_{\text{0}}\text{\textasciitilde{}}\;\text{half-Cauchy}\left(\text{0,}\;\;\text{2.5}\right)\;{\text{σ}}_{\text{1}}\text{\textasciitilde{}}\;\text{half-Cauchy}\left(\text{0,}\;\;\text{2.5}\right)$$

where p_si_ is a vector of yearly probabilities of bloom occurrence for location s; s∈1,…, 25, ${\text{X}}_{1},\ldots ,{\text{X}}_{4}$ are the fixed effects; and β_0_, …, β_4_ are the associated regression coefficients (Table 2). Model-predicted log odds for each location and year are back-transformed to probabilities for interpretation of results. The predicted probabilities are calculated using the inversion:

(2) $${\text{p}}_{\text{s}}=\frac{{\text{e}}^{\text{logit}\left({\text{p}}_{\text{s}}\right)}}{1+{\text{e}}^{\text{logit}\left({\text{p}}_{\text{s}}\right)}}$$

The prior parameter distributions are chosen to be non-informative or weakly informative. The Cauchy distribution is a common choice for a non-informative prior because it has support over the whole real line, with infinite mean and variance [50]. The 5th and 95th percentiles of the Cauchy (0, 2.5) distribution are about 15.8 and 15.8. An effect of −15.8 on the log odds of bloom occurrence would indicate that an increase of 1 in the *maxratio* (with all other variables held constant) would increase the odds of occurrence by a factor of e^15.8^ or about 7 million. Similarly, an effect of −15.8 translates to a decrease of about 7 million. A similar argument applies to the choice of the half-Cauchy for variance parameters, which has positive support yet does not restrict the magnitude of the location effect. Markov chain Monte Carlo (MCMC) sampling was used to fit the model using R 3.5.1 [51], the STAN software and the associated R package, rstan v2.21.1 [52]. Three chains were run, with 8000 iterations each. The first 2000 iterations of each chain were discarded as warmup. Initial values for each chain were obtained from the preliminary mixed effects modeling exercises.

#### 2.4.3. Persistence Model

A second model was developed in response to the experiences gained while trying to monitor and manage risks posed from the second cyanoHAB occurring in 2019. We realized then that the occurrence model lacked utility when a bloom was actively occurring. We desired a predictive model that could produce a probability of bloom conditions persisting: the persistence model. The responses of the persistence model are daily summaries of whether a bloom is occurring or not at each location for the 100 days following the maximum ratio day. Responses are assumed to follow a Bernoulli distribution, and the model is defined with a logit link function. The logit is the logarithm of the odds of bloom persistence where odds are defined by the probability of bloom persistence divided by the probability of bloom absence $\left(\frac{\text{p}}{1-\text{p}}\right)$. The following Bayesian model is fit:

(3) $$\text{logit}\left({\text{p}}_{\text{s}}\right)={\text{α}}_{0\text{s}}+{\text{β}}_{0}+\left({\text{α}}_{1\text{s}}+{\text{β}}_{1}\right){\text{X}}_{1}+{\text{β}}_{2}{\text{X}}_{2}+{\text{β}}_{3}{\text{X}}_{2}^{2}+{\text{β}}_{4}{\text{X}}_{3}+{\text{β}}_{5}{\text{X}}_{4}+{\text{β}}_{6}{\text{X}}_{5}+{\text{β}}_{7}{\text{X}}_{6}\;{\text{b}}_{\text{0j}}\text{ \textasciitilde{} Normal }\left({\text{0, σ}}_{\text{0}}\right)\;{\text{b}}_{\text{1j}}\text{ \textasciitilde{} Normal }\left({\text{0, σ}}_{\text{1}}\right)\;{\text{β}}_{\text{0}}\text{\textasciitilde{}}\;\text{Cauchy}\left(\text{0,2.5}\right)\;{\text{β}}_{\text{1}}\text{\textasciitilde{}}\;\text{Cauchy}\left(\text{0,2.5}\right)\;{\text{β}}_{\text{2}}\text{\textasciitilde{}}\;\text{Cauchy}\left(\text{0,2.5}\right)\;{\text{β}}_{\text{3}}\text{\textasciitilde{}}\;\text{Cauchy}\left(\text{0,2.5}\right)\;{\text{β}}_{\text{4}}\text{\textasciitilde{}}\;\text{Cauchy}\left(\text{0,2.5}\right)\;{\text{β}}_{\text{5}}\text{\textasciitilde{}}\;\text{Cauchy}\left(\text{0,2.5}\right)\;{\text{β}}_{\text{6}}\text{\textasciitilde{}}\;\text{Cauchy}\left(\text{0,2.5}\right)\;{\text{β}}_{\text{7}}\text{\textasciitilde{}}\;\text{Cauchy}\left(\text{0,2.5}\right)\;{\text{σ}}_{\text{0}}\text{ \textasciitilde{}}\;\text{half-Cauchy}\left(\text{0,2.5}\right)\;{\text{σ}}_{\text{1}}\text{\textasciitilde{} half-Cauchy}\left(\text{0,2.5}\right)$$

where p_s_ is a vector of daily probabilities of bloom occurrence for location s. ${\text{X}}_{1},\ldots ,{\text{X}}_{6}$ are the fixed effects, and β_0_, …, β_7_ are the associated regression coefficients (Table 2). Predictors unique to the persistence model are X_2_, which represents the number of days (0, 1, 2, …, 100) after the date on which the maximum ratio occurred, and X_3_, which is a binary indicator of a sharp increase in flow (1 if the 1–19 day lagged average exceedance has decreased by more than 15 in the previous 19 day, and 0 otherwise), providing a mechanism for bloom cessation. The selection of 15 and 19 was based on the data during the end of the 2015 and 2019 blooms during which a decrease of this magnitude was observed at almost all locations. Boundary conditions and prior distributions are the same or similar to the occurrence model. As above, MCMC sampling was used to fit the model with four chains and 6000 iterations each. The first 2000 iterations of each chain were discarded as warmup, and the initial values were again obtained from a preliminary mixed effects model. Modeling assumptions are discussed, and convergence statistics are provided in Supplemental Materials and Tables S1 and S2 and Figures S3–S8.

### 2.5. Water Quality Data Visualization

Although WQ data were not used directly in the model development, we incorporated visualization and download options for eight sites currently reporting continuous WQ data into the same application meant to serve the results of the occurrence and persistence models. It varies among sites, but WQ variables generally include water temperature, dissolved oxygen, specific conductance, and pH, among others and depending on the site. More details on the characteristics of the available WQ data are provided in supplemental materials. We had three objectives for the WQ visualization component of the risk characterization tool: users should be able to (1) compare data across sites; (2) compare data across years at a site; and (3) plot daily differences of at least two WQ variables. The latter capability can prove an indicator of the degree of algal activity. To make this integration and visualization possible, data needed to be retrieved from different host locations (Table S3), and visualization strategies were developed as demonstrated below.

### 2.6. R Programming and Risk Characterization Tool

Statistical analyses and risk characterization tool development were completed using the R programming language [51]. R software was used to support data acquisition, statistical modeling phases, and development of the R Shiny app for this effort. Shiny is an open-source R package that provides a framework for building web applications using R (https://shiny.rstudio.com/ accessed on 23 June 2020). The risk characterization tool is a Shiny app that was developed under a framework of (1) providing a platform for serving the risk probability modeling results and housing supplementary information related to the modeling effort, which is meant to be updateable through time; and (2) serving as a source for acquiring and visualizing a variety of different water data types from multiple sites and multiple years simultaneously in support of real-time water quality management operations.

## 3. Results

### 3.1 Supporting Evidence for Key Drivers in the Conceptual Model

We include nutrient loads estimated from the ORSANCO data in tributary and main river sites collected during the month of July among 16 years of monitoring as supporting evidence of the supposition that excess nutrients in the river in the period just before the development of the bloom in 2015 are potentially an important controlling factor for cyanoHABs (see Figure S9). Total Nitrogen (TN) load from the tributaries of the upper river was higher in July of 2015 than the previous 13 years. At mid-river, the TN and total phosphorus (TP) loads for 2015 rated in most cases as the highest observed since the nutrient monitoring program began.

Following the period of high nutrient loading, the postulated requisite period of low flows begins, which results in higher pool residence time and opportunity for the cyanobacteria biomass to build to a bloom condition. We offer supporting evidence for the supposed importance of residence time by plotting estimates for L&Ds in the upper river (see Figure S10). As the low flow conditions began toward the end of July in 2015, residence times rose more sharply in the pools of the downstream sites that bloomed in 2015, with a potential threshold suggested of ca. 3 days.

### 3.2. Occurrence Model Results Demonstration

**Figure 5.**
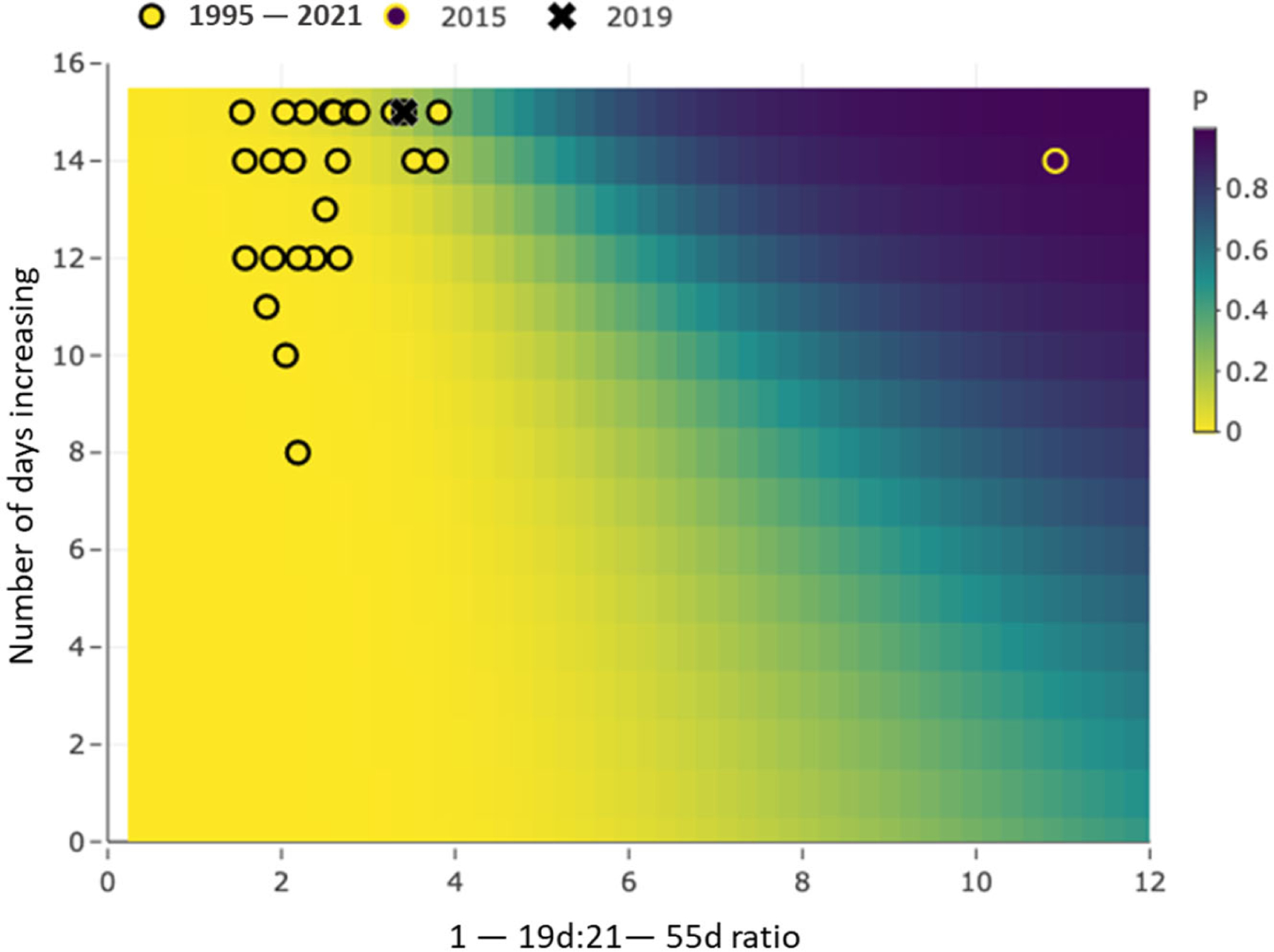
Graphical bloom occurrence model results for the Greenup site. Data are yearly 1–19-day:21–55-day *maxratios* and number of days increasing (*inc15s*) for the bloom season overlaying gradient in predicted risk probabilities (P) at right. CyanoHABs in 2015 and 2019 years are identified per the legend at the top.

Model inputs for the bloom occurrence model are location, the current day’s lagged exceedance ratio, and the number of days the ratio has been increasing. Model output includes the probability that the current year will be a bloom year with 95% credible interval. We plot the model results for bloom probability at the Greenup site in Figure 5 as an example and with the following interpretation: If a user observed 15 August 2019 a current-day lagged exceedance ratio of, say, 3.7 that had been increasing for the last 15 days, then the model predicts a 23% probability of a bloom occurring at the site in 2019 with a 95% credible interval of 1.7 to 68%.

**Figure 6.**
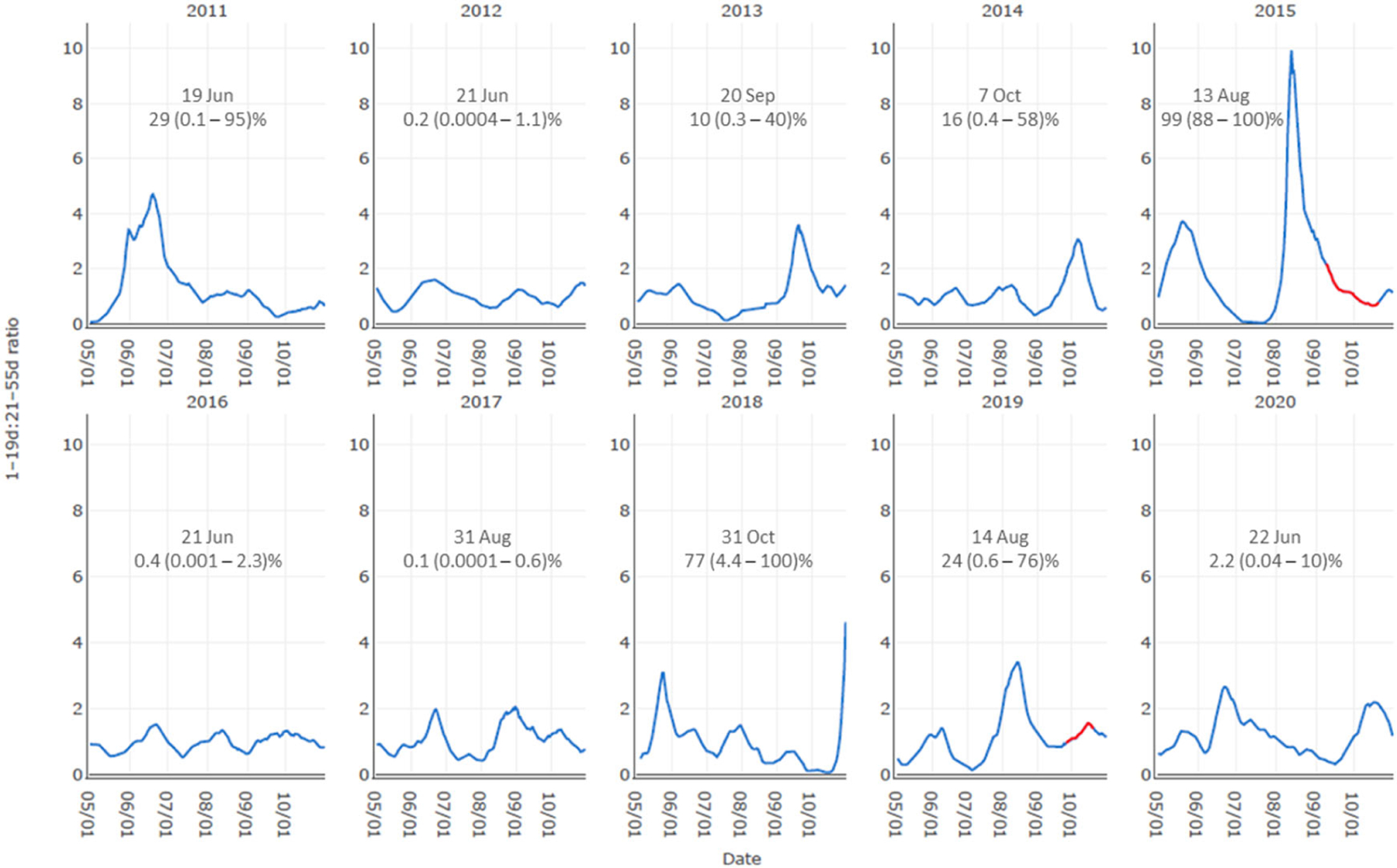
Daily 1–19day:21–55 day lagged exceedance ratio at Markland Site plotted for each year from 2011 through 2020. Text in each graph denotes the day that the *maxratio* occurred in each year and what the occurrence model’s prediction probability would have been with the 95% credible interval in parentheses. Data in red are the documented bloom periods.

The limited number of bloom events constrained formal model validation but results for bloom probabilities on the day *maxratio* was observed for a 10 year period at the Markland site, which experienced a cyanoHAB in both 2015 and 2019, suggested the 2019 bloom was well-predicted (Figure 6). In addition to these years, notable cyanoHAB risk probabilities (i.e., above 20%) occurred in 2011 and 2018, but in each of these cases, the credible interval was large, and they occurred earlier and later in the season, respectively, and likely outside of the window of opportunity for cyanobacteria to develop even though flow conditions were favorable. Leave-one-out cross validation to evaluate the occurrence model performance produced a misclassification rate of 2.8% (refer to Supplemental text and Figure S7).

### 3.3 Persistence Model Results Demonstration

**Figure 7.**
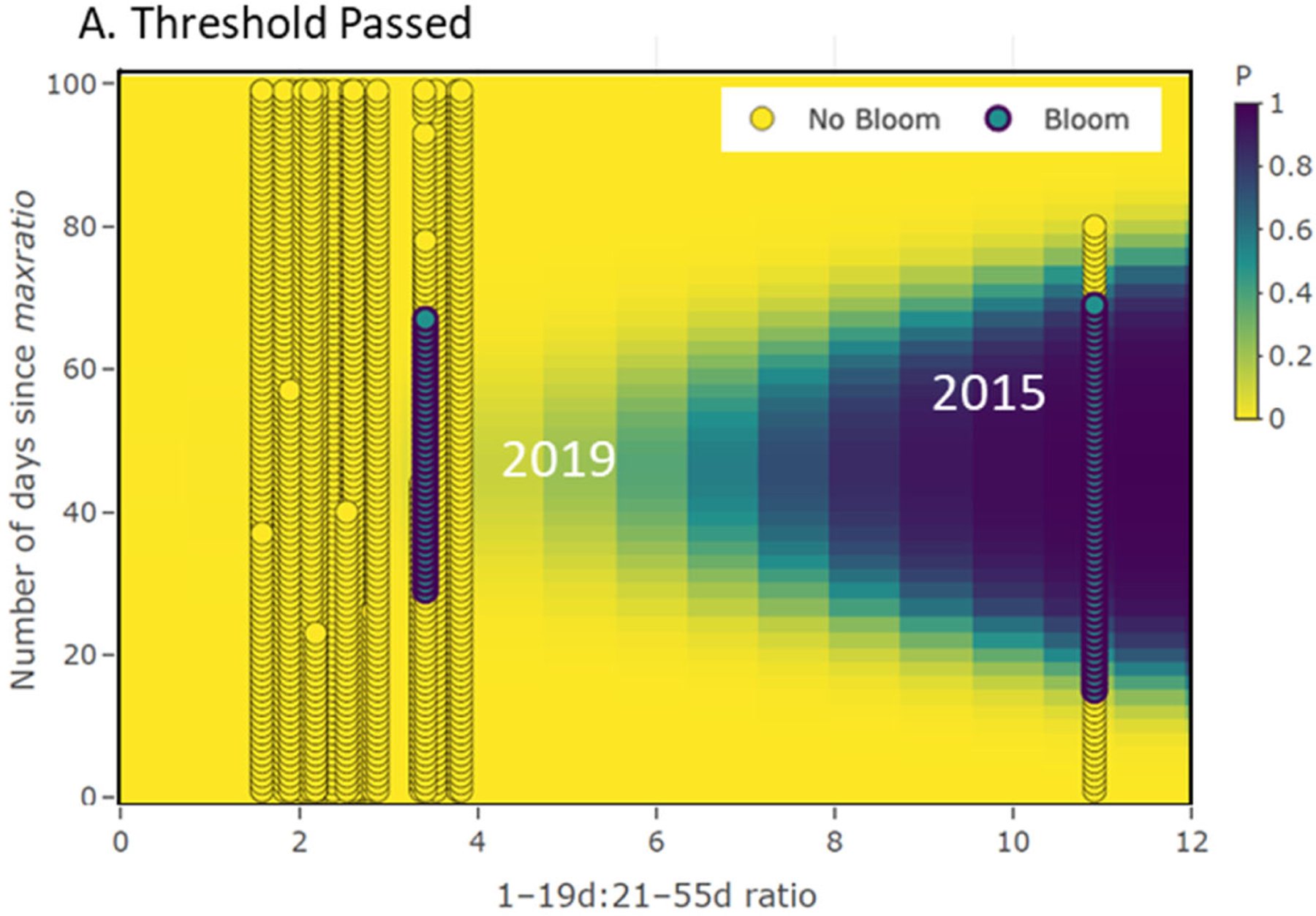

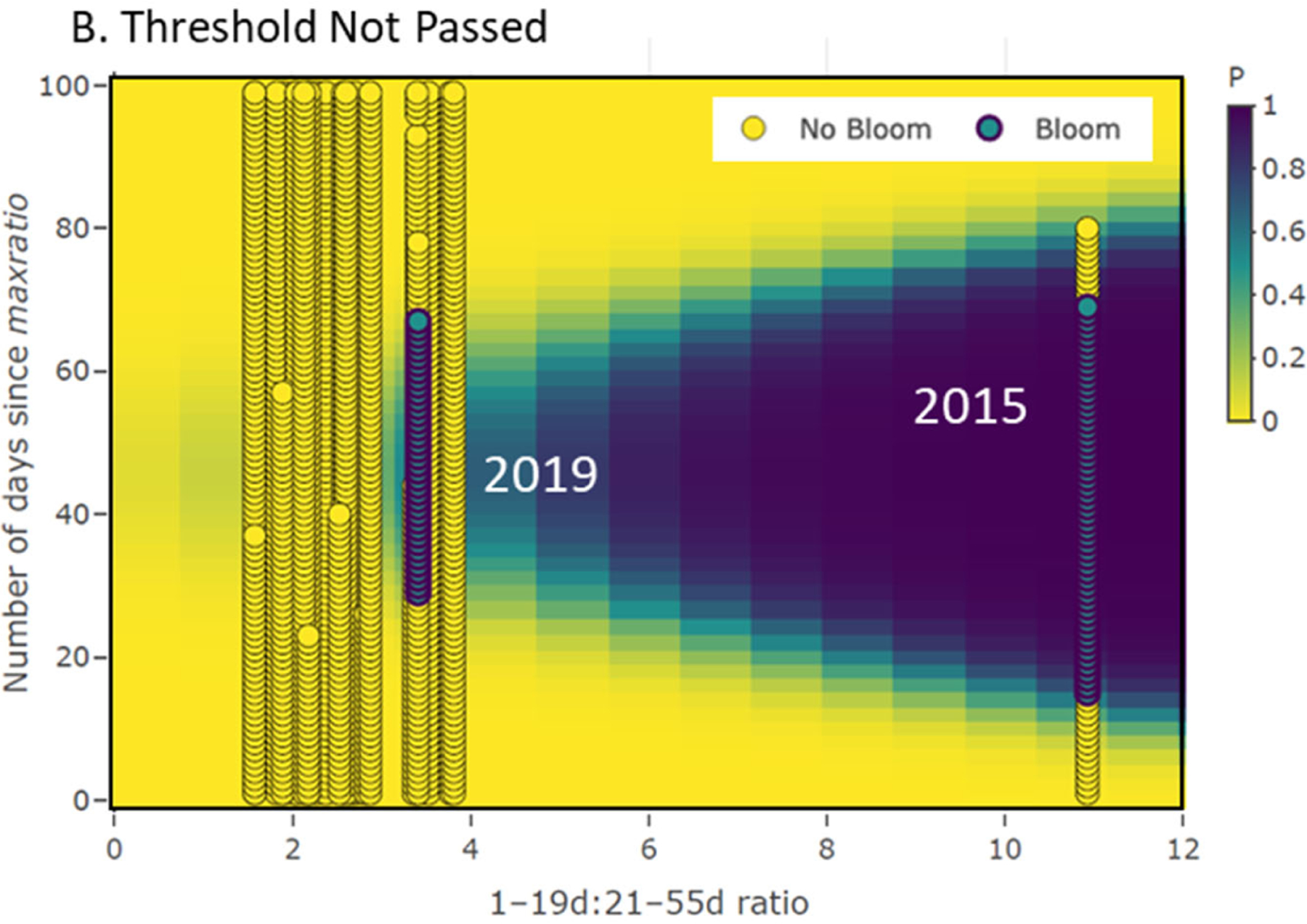
Yearly *maxratio* for the bloom season computed for the Greenup site plotted vs. the number of days since the *maxratio* occurred for each of the 25 years modeled. Each vertical line of points represents a year. Differences between (**A**,**B**) demonstrate how persistence probability increases if the threshold indicator has not been passed (i.e., cone of high probability shifts to the left).

With the quadratic structure of the persistence model, the resulting risk probability prediction is conditioned on the number of days since the *maxratio* for the year has passed at the site for the year predicted. For periods near the day the *maxratio* is observed, the risk of bloom persistence is lower, as are the cases for days further out. Therefore, there is a cone shape to the prediction probability with the highest risk occurring at intermediate times (days) from when the *maxratio* occurred (Figure 7). Operationally, the model’s threshold indicator term effectively lowers the risk of a bloom occurring if flows have been increasing (Figure 7A).

The leave-one-out cross validation of the persistence model had a misclassification rate of 3.4% (Supplemental Materials text and Figure S8). The risk interpretation of the persistence model output is based on the relative proximity the current day of prediction is to the day the *maxratio* occurred. With Figure 7 as an example, a *maxratio* of 3.68 occurred on 8/15 at the Greenup site in 2019. If it was 50 days after the day *maxratio* occurred and the threshold indicator had not been passed, then the probability of a bloom persisting at this site was close to 50% (whether one had occurred or not: in this case, it had). In contrast, had we estimated the probability 25 days earlier or later, the probability of persistence would have dropped to zero.

### 3.4. Shiny App Real-Time Reporting

**Figure 8.**
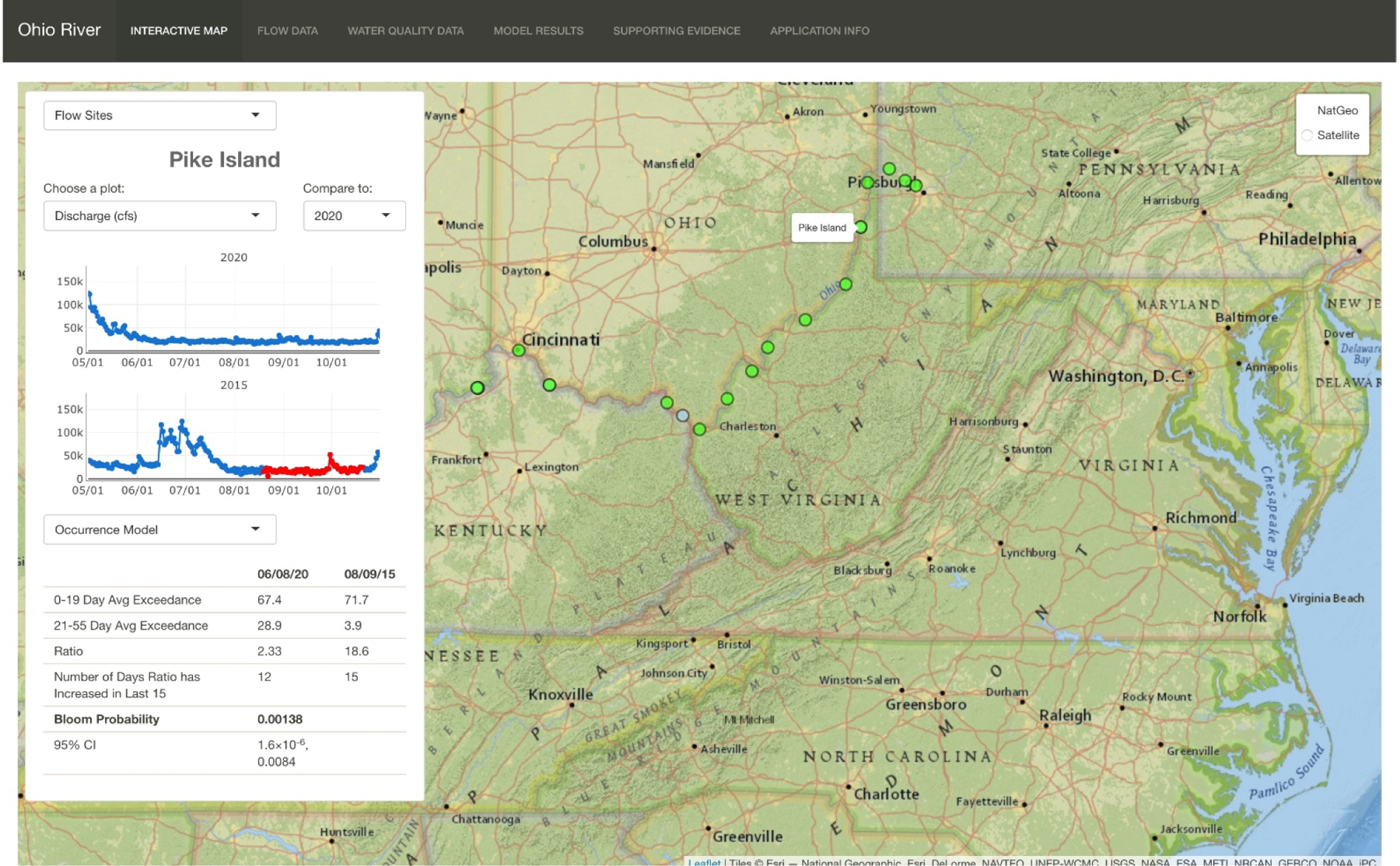
Screen capture of interactive map page of the risk characterization tool. In this image, the Pike Island site has been selected, and discharge data are reported for 2020 (a non-bloom year) compared to 2015 (a bloom year). The results of the lagged exceedances are computed for the day in 2020 that the *maxratio* occurred and the bloom probability predicted by the occurrence model for this year. In real time, during the bloom season, the flow series would be up-to-date, and the model results would be reported for the most current date that flow had been reported for the site.

The results of the Shiny app development effort are presented as screenshots (Figures 8 and S11–S13). The Shiny app is currently accessed through the ORSANCO website at (https://orsanco-hab.shinyapps.io/shiny-ohio-river/# accessed on 29 December 2021). The landing page is an interactive map that serves model results in real time (Figure 8). The functionality of the app is dependent on data that are scraped from websites of other organizations (see supplemental text and Table S3). There is an embedded window that allows users to make site selections and configure data plots. Sites on the entry map are color-coded based on the results of the occurrence model for the current day. Current data from the sites with active water quality sensors can also be studied. Finally, the header ribbon allows the user to select visualization and download options for “Flow Data”, “Water Quality Data, “Model Results”, “Supporting Evidence”, and “Application Info”.

The Flow Data tab offers tabular data or plots of historical flow data at a site and functions for interacting with this information. A graph view “grid” option presents flow data in the manner of Figures 3 and 4, whereas a “stacked” option offers an interactive time series plot with multiple years of data (Figure S11).

The Water Quality Data page offers three display options: tabular data, site comparisons, and year comparisons. The latter two were designed to provide spatial and temporal context for current year trends relevant to the active monitoring of cyanoHAB events. With the site comparison display, one or two variables can be visualized at the same time across multiple sites, as can the trend in differences between daily maximum and minimum values (refer to supplemental text associated with Figure S12). The “Year Comparison” display allows users to visualize and interact with data from multiple years for the same site (Figure S13 and associated supplemental text explanation). The app design for the WQ visualization approach is geared toward indicators of bloom conditions at different locations along the river or determining if WQ trends are suggestive of bloom conditions when the occurrence or persistence models are returning higher risk probabilities. Hence, the WQ options in the app potentially offer a means of validating the risk modeling results at certain sites. Likewise, the year comparison display is useful for evaluating whether the current year’s water quality data are responding in a similar fashion as previous years.

## 4. Discussion

Our approach to the development of the cyanoHAB risk characterization tool evolved from three primary conditions: (1) the assumed limitations posed by the fact that only one or two bloom events had occurred on the river in the past at any of the data reporting locations; (2) the limited availability of data directly linked to the phytoplankton community at any site; and (3) the expressed need of the end users to be able to assess the risk of cyanoHABs over the entire river at once and in real time. These drove the predictive modeling to focus on the interpretation of historical flow dynamics and the configuration of the Shiny app that reports prediction probabilities with additional diagnostics for interpreting the water quality from the limited number of sites with continuously reported data. Here, we offer the scientific rationale for why we consider this a sound approach for handling cyanoHAB risk characterization in a regulated river like the Ohio.

First, much of the research on cyanoHAB forecasting to date has benefited from satellite-based data. Good examples of this in the United States come from the Western Basin of Lake Erie [53,54] and the CyAN app that offers data on bloom condition from over 2000 moderate to large-size lakes [47,55]. However, the width of the Ohio River was a barrier to using current satellite data to bolster the risk characterization.

Second, while direct measurements of the phytoplankton community are invaluable to assessing community structure and the presence of toxin producing taxa, these data come from a grab sampling effort that requires a physical presence to take the sample and have processing times that can range from several hours to many days before getting the results. Nevertheless, we did consider opportunities for incorporating grab sampling data into our framework more directly. As described in supplemental text, ORSANCO’s large repository of mainstem phytoplankton taxonomy data did not prove beneficial to identifying past bloom events that could have been used for model fitting. We also considered the potential relevance of allochthonous taxa inputs as predictors of mainstem blooms, reasoning that, if important, data from some of the more heavily sampled tributaries and reservoirs therein may be useful to making predictions on the river’s mainstem. However, two Ohio River studies and one focusing on the Kansas river suggested that mainstem algal community dynamics tends to be independent of tributary inflows during low flow periods [56–58], respectively. This led to the thinking that strategic low-flow mainstem monitoring may yet still prove useful, which was tested during the 2019 bloom at a site in the Markland pool near ORSANCO’s headquarters where the phytoplankton community was assessed via microscopy counts on more frequently collected grab samples (see Figure S14).

The results suggested that, even under low flow conditions, the river hydraulics imparted variability that would constrain the utility of grab sampling for predictive modeling. While cyanobacteria dominated the community coincident with the timing of the Markland bloom report in 2019 (Table 1), they had returned to pre-bloom relative abundances over the next two weeks of bloom persistence (Figure S14). Furthermore, the taxonomic assessment of these samples showed that the toxin producers *Planktothrix* and *Microcystis* were the most abundant among the cyanobacteria, but two weeks later, while the bloom toxicity remained high, neither genus was observed. Then a week after this, *Microcystis* was again a community dominant (see Figure S15). This temporal variability in direct cell counts from a single site indicated to us that it would make little sense to base modeling effort on direct measures of cyanobacteria abundance, even if they had been available for multiple sites along the river. Indeed, this dimension of the cyanoHAB risk management problem stimulated the interest in developing the persistence model, whose flow-based predictions are meant to inform river sampling strategies when choosing one location vs. another to sample for toxins can mean the difference of several hundred kilometers.

Third, we considered the in situ sensing of chlorophyll and phycocyanin pigments as more direct and time relevant indicators of cyanobacteria biomass [59]. Indeed, fixed location phytoplankton pigment sensing has been used with some success for signaling and forecasting risk related to cyanoHABs [59–64]. This strategy can work well when there are specific points of public health concerns such as drinking water intakes or swimming beaches. Chaffin, et al. [65,66] have studied the performance of these sensors along with the suite of other sensed water quality variables that typically accompany the pigment signals. They caution that sensor placement guided by stakeholder-specific criteria can render the information ineffective for predictive modeling.

As a case in point, we can demonstrate such ineffectual in situ sensor data from a suite deployed in the Greenup pool on the Ohio River. The data from the sensors produced ambiguous results on bloom status during the 2019 cyanoHAB, with the in situ signals of both chlorophyll and phycocyanin suggesting no indications of increasing or excess phytoplankton biomass prior to or during the period of the bloom (see Figure S16). This lack of sensor response was likely due to significant spatial heterogeneity within the pool. In fact, such spatial heterogeneity was mapped both longitudinally and vertically through the water column during a survey conducted in the Markland pool, downstream of Greenup, during the 2019 cyanoHAB event (see Figure S17). Figuring out how to best incorporate such degrees of both temporal and spatial variability directly into a predictive modeling framework and over such distances as the length and width of the Ohio River is daunting. Instead, the ever-growing availability of continuously sensed water quality data were incorporated into our risk characterization framework in the form of the diagnostic visualizations built into the Shiny app.

Finally, while rare, we note that there are a few examples of cyanoHAB prediction models developed for other regulated river systems outside of the United States. Primary examples come from hypereutrophic rivers in South Korea and China, e.g., [39,67–69] and [40,70–73]. Each of these studies benefited from the availability of sufficiently dense water quality data coincident with bloom events. However, none were intent on providing predictions for sites spanning such a length of river compared to our study. Furthermore, in common among these studies was the significance of flow (or aspects thereof) as a predictor, while there was a lack of consistency among them in terms of the significance of other predictors such as nutrients and/or other general water quality variables, including temperature and turbidity.

The results from these studies provide supporting evidence that low flow conditions must be of long duration for cyanobacteria to concentrate to a bloom status on a large, regulated river. However, unique to our treatment was the use of the historical flow time series data and accounting for the magnitude and timing of the preceding high flow conditions that was necessary to fit a significant model. We hypothesized that the controlling mechanism behind the significance of the high flow period had to do with nutrient bioavailability. Even though average nutrient concentrations from grab sampling suggest non-limiting conditions for phytoplankton growth in the Ohio River (see Figure S18), we know that typical approaches to grab sampling or in situ sensing would not capture the reduced forms of nitrogen that can be a significant driver of blooms in lakes [14,74]. We suspect that the large loads of particle-bound nutrients that come with the high flow events are being detained behind the river’s flow control structures as flows decrease. The trapped nutrient loads are then made more available by the hydrodynamics of the pool shifting to more stratification-like conditions that, in turn, affect nutrient biogeochemistry, as described in Smucker, Beaulieu, Nietch and Young [29]. With planktonic cyanobacteria able to control their position in the water column with gas vacuoles, nutrient-rich deeper waters become more accessible [26]. We suggest this plausible series of ecosystem state transitions to help explain why the timing of the high flow period relative to the low flow period appears so critical to bloom development.

With the real-time flows among 25 sites used to derive the main predictors in the occurrence and persistence models coupled with the configuration of the visualization options for the continuous water quality data, the Ohio River cyanoHABs risk characterization tool provides a sound framework for assessing river conditions related to cyanoHAB ecology. The content organization of the Shiny app was based on the practices used by water quality managers to make decisions about when and where samples should be taken and to communicate potential risks of river conditions to stakeholders. When a user arrives at the Shiny app’s opening page, they can determine the bloom occurrence probability of all sites simultaneously. Clicking on a site of interest adjusts the embedded data window and provides options for visualizing data trends. If a site on the entry page returns a concerning bloom prediction, the “Water Quality” tab can be selected to visualize recent trends at the site and relative to what is being reported at other sites or has been observed in previous years from the same site. These comparisons offer additional diagnostics for gauging risk and help to inform appropriate management actions. Finally, using the Shiny app offers river managers a means of studying river conditions within the context of factors known to be important to cyanoHAB dynamics. This fosters experiential learning that can be used to guide updates and tool improvements over time.

## Supplementary Material

Supplementary File

## Figures and Tables

**Table 1.** Ohio River sites where flow is estimated on a continuous basis along with the cyanoHAB reporting dates for the 2015 and 2019 events. L&D = Lock and Dam. NA = not applicable for model development.

#	Site Name	Latitude	Longitude	Type	River Miles below Pittsburgh, PA	2015 HAB (Date First Observed)	2019 HAB (Date First Observed)	Used for Modeling (+)| Model Results Reported (✔)
1	Pittsburgh	40.43944	−80.01083	Mid-Pool	0	No Bloom	No Bloom	NA
2	Emsworth	40.50500	−80.08972	L&D	6.2	No Bloom	No Bloom	✔
3	Dashields	40.54972	−80.20694	L&D	13.3	No Bloom	No Bloom	✔
4	Montgomery	40.64722	−80.38889	L&D	31.7	No Bloom	No Bloom	✔
5	New Cumberland	40.52806	−80.62583	L&D	54.4	No Bloom	No Bloom	✔
6	Pike Island	40.14972	−80.70167	L&D	84.2	19 August 2015	No Bloom	✔
7	Hannibal	39.66722	−80.86611	L&D	126.4	21 August 2015	No Bloom	✔
8	Willow Island	39.35900	−81.32400	L&D	161.7	24 August 2015	No Bloom	✔
9	Marietta	39.40944	−81.45778	Mid-Pool	172	24 August 2015	No Bloom	+
10	Parkersburg	39.26806	−81.56389	Mid-Pool	185	24 August 2015	No Bloom	+
11	Belleville	39.11800	−81.74200	L&D	203.9	24 August 2015	No Bloom	✔
12	Racine	38.91800	−81.91100	L&D	237.5	25 August 2015	No Bloom	✔
13	Point Pleasant	38.84389	−82.13972	Mid-Pool	265	26 August 2015	No Bloom	+
14	RC Byrd	38.68000	−82.18500	L&D	279.2	27 August 2015	No Bloom	✔
15	Huntington	38.41333	−82.50056	Mid-Pool	312	27 August 2015	12 September 2019	✔
16	Ashland	38.48111	−82.63667	Mid-Pool	322	27 August 2015	11 September 2019	+
17	Greenup	38.64667	−82.86056	L&D	341	27 August 2015	12 September 2019	✔
18	Mayesville	38.68389	−83.78389	Mid-Pool	409	28 August 2015	12 September 2019	+
19	Meldahl	38.79722	−84.16667	L&D	436.2	1 September 2015	17 September 2019	✔
20	Cincinnati	39.09444	−84.51056	Mid-Pool	471	9 September 2015	19 September 2019	✔
21	Markland	38.77472	−84.96444	L&D	531.5	9 September 2015	26 September 2019	✔
22	McAlpine	38.28028	−85.79917	L&D	606.8	11 September 2015	24 September 2019	✔
23	Cannelton	37.89944	−86.70556	L&D	720.7	15 September 2015	No Bloom	✔
24	Newburgh	37.92833	−87.37500	L&D	776.1	16 September 2015	No Bloom	✔
25	Evansville	37.97222	−87.57639	Mid-Pool	792	17 September 2015	No Bloom	✔
26	John T. Meyers	37.78333	−87.97944	L&D	846	18 September 2015	No Bloom	NA
27	Smithland	37.15833	−88.42611	L&D	918.5	19 September 2015	No Bloom	✔

**Table 2.** Explanation of parameter in the cyanoHABs predictive models.

Parameter	Variable Name	Effect	Description
Occurrence Model			
X_1_	*maxratio*	fixed	maximum 1–19:21–55 day ratio
X_2_	*inc15*	fixed	Number of days increasing in 15 days prior to *maxratio* day
X_3_	*meanrt*	fixed	Site’s mean residence time
X_4_	*maxratio × meanrt*	fixed	interaction term
α_1_	na	random	*maxratio* slope adjustment
α_0_	na	random	*maxratio* intercept adjustment
β_0_, …, β_4_	na	na	regression coefficients
Persistence Model			
X_1_	*maxratio*	fixed	maximum 1–19:21–55 day ratio
X_2_	days	fixed	Number of days after *maxratio*
X_3_	binary indicator (1 or 0)	fixed	indicator of increase in flow
X_4_	binary indicator × *maxratio*	fixed	interaction term
X_5_	meanrt	fixed	site’s mean residence time
X_6_	*maxratio × meanrt*	fixed	interaction term
α_1_	na	random	*maxratio* slope adjustment
α_0_	na	random	*maxratio* intercept adjustment
β_0_, …, β_6_	na	na	regression coefficients

na = not applicable.

## Data Availability

Data supporting the model development can be downloaded using the Shiny app (https://orsanco-hab.shinyapps.io/shiny-ohio-river/, accessed on 29 December 2021) or are available through the USEPA’s Science Inventory web interface (https://cfpub.epa.gov/si/, accessed on 29 December 2021).
