# Supplementary material for: Development of a Risk Characterization Tool for Harmful Cyanobacteria Blooms on the Ohio River
Source: Water (Basel). Author manuscript; Available in PMC 2022 Apr 20. (PMC9019831; doi:10.3390/w14040644)

Ohio River | Interactive Map | Flow Data | Water Quality Data | Supporting Evidence | Application Info

Select a display:

Year Comparison

Select a site:

Markland

Select a parameter:

Dissolved Oxygen (mg/L)

This is an interactive plot.
Hover over the plot, and use the icons to interact with it.
Use the magnifying glass icon to draw a zoom box.
Use the house icon to reset the axes of the plot.
Select any combination of legend elements by clicking on them.

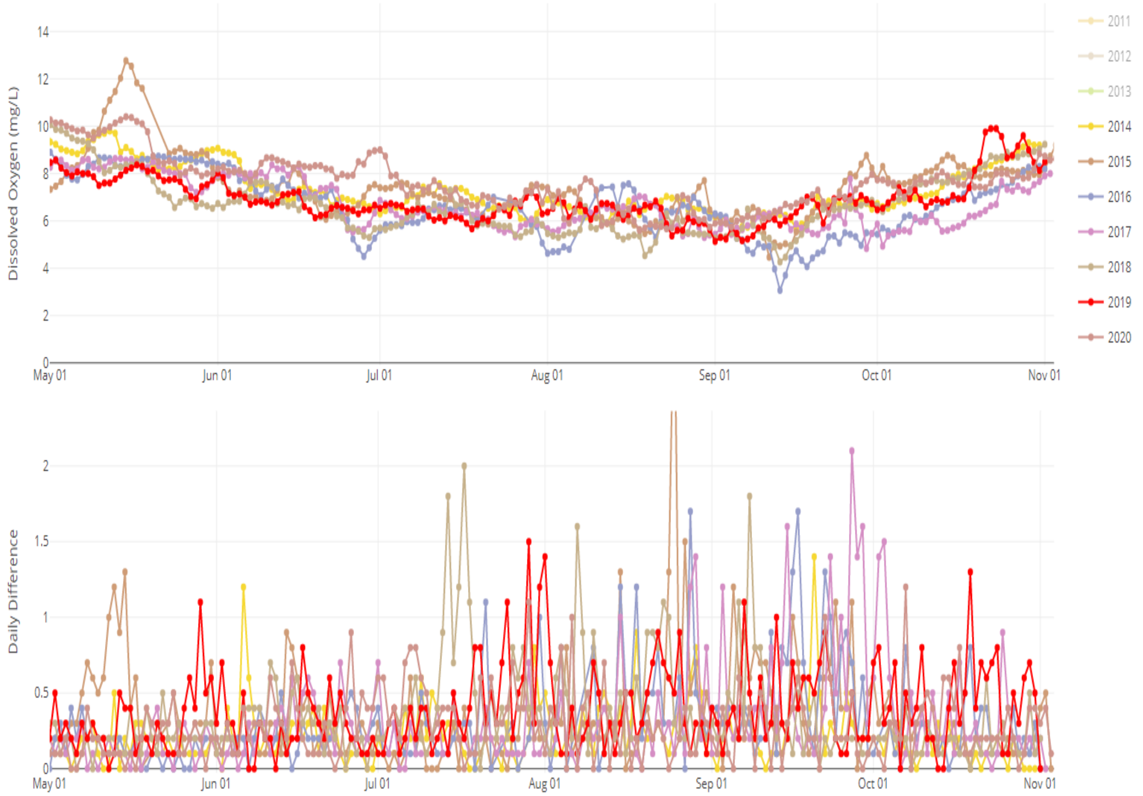

Supplement: Supplementary File [file NIHMS1783469-supplement-Supplementary_File.zip › water-1558146-supplementary-proof done/FigureS13.pdf]
