# Supplementary material for: Development of a Risk Characterization Tool for Harmful Cyanobacteria Blooms on the Ohio River
Source: Water (Basel). Author manuscript; Available in PMC 2022 Apr 20. (PMC9019831; doi:10.3390/w14040644)
Supplement: Supplementary File [file NIHMS1783469-supplement-Supplementary_File.zip › water-1558146-supplementary-proof done/Supplemental Text.pdf]

Ohio River Hydrography and Water Data Sources and Treatment

The Ohio River is regulated with a series of locks and dams (L&D). The USACE operates the L&D system on the river (Figure S1, https://www.lrd-wc.usace.army.mil/OhioRiver/OhioRiver.html, accessed on 10 October 2018). The sections of river between L&Ds are referred to as pools, with the pools named by river hydraulic managers after the name of the downstream L&D that forms the pool. For example, the Markland pool identifies the river section between the Captain A. Meldahl L&D and the Markland L&D.

For data related to river flows, the USGS operates a comprehensive network of gauging stations in the Ohio River basin including mainstem river stations that are accessible through their National Water Information System: Web Interface (https://waterdata.usgs.gov/nwis, accessed daily). A search for sites reporting gauge height and river discharge returns 38 and 12, respectively. However, continuous, long-term records of measured discharge are limited to only two locations: an upper river site, the Dashields L&D, and a down river site at Shawneetown, IL. Furthermore, there is a lack of consistency among stations reporting observed stage and calculated discharge in terms of when data reporting started: Two of the stations reporting gauge height go back to the 1980s, ten offer data beginning in 2010, eight others became available in 2014, and six others in later years. Five stations report discharges back to the 1930s and 1940s.

Many of the USGS sites are maintained under contract with the USACE at L&Ds. For each L&D, gate and tailwater flow rating curves were developed after dam construction. Among the active USGS sites sensing gauge height at L&Ds, the rating curves are used to estimate current flow conditions. For the past, The Lakes and Rivers Division of the USACE has made available digital datafiles of manual observations of pool level, tailwater level, total gate opening, and hydropower flow (if appropriate) at each L&D since 1995 (http://www.lrd-wc.usace.army.mil/OhioRiver/OhioRiverNavData.html, accessed on 10 October 2018). These data are typically recorded in one-hour intervals, with increased frequency during rapidly changing conditions.

The OHRFC (url: https://www.weather.gov/ohrfc/ accessed on 20 October 2018) currently uses real-time monitoring from these USGS and USACE sources to provide model guidance and support for drainages upstream of Smithland L&D in the Ohio Basin and using a suite of hydrologic, hydraulic, and related forecasting models. These models provide river stage and flow forecasts at 54 sites to help support flood control and navigation. They are generally calibrated with a focus on forecasting flood conditions (or high flows) but are used daily to forecast stages across the full range of flows. The same techniques OHRFC has used to provide discharge data in its routine forecasting operations were used here to reconstruct synchronized time series of discharges beginning in 1995 and that currently report river stage in real time.

Within the L&D system, the backwater influence of each dam structure decreases upstream and is a minimum at the tailwater of the next dam upstream. For L&Ds, computation of discharge depends on the flow rate at each structure. When the dam is "in pool" (i.e., flow is insufficient to support the federally regulated 11′ depth for navigation throughout the pool, and the dam is needed to control pool depth), discharge is computed by a gate-rated

flow and with the addition of total reported hydropower discharge where appropriate. When river flow increases to the point where the dam is not necessary to support navigation, gates at the L&D are raised above the water surface, and the river changes into a free-flow condition. This free-flow threshold is evident from an example of the upper pool and tailwater stages to discharges estimated from gate ratings at the Meldahl L&D (Figure S2a,b). On the downstream side of the dam, the tailwater (Figure S2b), the correlation between stage and discharge, suggests relatively limited hydraulic influence from the next L&D or other tributaries entering downstream. These influences become more apparent at the Cincinnati stage gauge (Figure S2c), were the correlation, while strong, contains more scatter, especially under lower stages, indicating the impact of increasing proximity of Markland L&D, as well as the influence of several tributaries entering between the Meldahl L&D and Cincinnati. This analysis applied at each of the 27 sites with stage data disqualified upper pool stations and eliminated one L&D entirely and two mid-pool sites.

For water quality data, we started with ORSANCO's repository of phytoplankton cell counts data with an objective of identifying periods in the past where blooms may have occurred but had gone unreported or had not received attention as a potential risk to public health. This was spurred by the reports of the large bloom in 2008 in the vicinity of Cincinnati, OH. More than 1500 samples from 12 stations covering 13 years were reviewed, only to find five observations with cyanobacteria cell densities above 20,000 cells/mL and with a maximum slightly above 52,000 cells/mL among them; the 2008 bloom event at Cincinnati was not evident. With cell counts up to 31×10^6 cells/mL recorded during the 2015 cyanoHAB event, we determined that there was no direct evidence of significant cyanoHAB events over at least the previous 13 years that could be used to support our modeling effort.

ORSANCO also conducts routine nutrient monitoring, visiting 35 sites (18 mainstem and 17 tributary sites) every other month (six times per year), starting in January 2000. Although this grab sampling data proved useful for characterizing inter- and intra-annual trends in river nutrient concentrations and loadings, we deemed the collection frequency too infrequent to be used directly in model development. Similarly, there proved to be little continuous and long-term data available for other water quality variables (i.e., in situ sensor data). While several drinking water utilities deploy sensors to monitor conditions of their raw water, in all cases that we were aware of, sensors were not in locations conducive for assessing river conditions that could be directly related to cyanoHABs occurring at or near the surface of the river. Therefore, we deemed it ineffective to try to compile and quality control such data from the DWTPs along the river.

Finally, as this research progressed, the number of sites reporting continuous water quality on the Ohio river increased from two to eight. The USGS reports data from two super gauge sites on the river, one reports publicly available data starting in 2013 near the mouth of the river at Olmstead, IL, and the other has data available since September of 2015 from a site near Ironton, OH. Two additional USGS sites report water quality from Markland and Cannelton L&Ds. The reporting history, number, and type of water quality measurements differs between these sites. Similarly, starting in 2019, ORSANCO began to establish four seasonal water quality monitoring

stations along the river at Pike Island, Meldahl, Markland, and Newburgh. These sites have sensors for measuring water temperature, pH, specific conductivity, dissolved oxygen, turbidity, phycocyanin, and total chlorophyll. The latter two measures are indicative of the relative concentration of cyanobacteria and total algae density, respectively. Data from many of these sites during both the 2015 and 2019 blooms were not available or lacked reporting consistency among them. This precluded their use in our model development.

Modeling Assumptions, Convergence, and Validation Statistics

The assumptions required for our modeling approach warrant some explanation. We assume linearity, which is difficult to assess empirically within a given location since there was only one or two years in which blooms occurred. However, we consider it reasonable relative to our conceptual model that as the *maxratio* or *inc15* increases, the log odds of bloom occurrence would increase by a proportional amount if all other variables are fixed. For the persistence model, a quadratic relationship is modeled between the log odds of bloom occurrence and the number of days after the *maxratio* was passed and assuming location, *maxratio*, and the threshold-passed-indicator are fixed. A site's mean residence time is incorporated in the models so that bloom probability depends both on residence time, the *maxratio*, and the *inc15*. After accounting for these three variables, independence across sites is assumed. This assumption is supported by the river managers who noted the failed attempt to use the Ohio River spill transport model [75] to track the timing of the 2015 bloom in the downstream direction. Similar inconsistencies were noted for bloom dynamics in the Kansas River, where measured concentrations of cyanobacteria and associated compounds indicated that simple dilution models were not sufficient to describe the downstream transport [58]. Regardless, the *meanrt* predictor was added to the model as a proxy for spatial location, given that farther upstream sites tend to have lower average residence times. In this way, spatial location is indirectly accounted for in the mean structure of the model, because sites closer together on the river tend to have similar *meanrt* and thus are similar in terms of bloom occurrence.

The responses are summarized yearly for the occurrence model to minimize temporal autocorrelation. Whether a bloom occurs from one year to the next can be viewed as functionally independent, although we realize that akinetes and overwinter potential exist from lake studies ([77,78]). For the persistence model, the responses are summarized daily, and the relationship between the response from one day to the next is accounted for by the number-of-days variable. Independence is still assumed from one year to the next. Unaccounted for binomial variation may be present, which may lead to underestimation of model uncertainty. However, this was not a major concern given that (1) features that may lead to it are not expected to be severe (i.e., violations in independence and model inadequacy), and (2) model uncertainty is already large due to the limited sample size under bloom conditions.

Convergence statistics (Table S1) and trace plots (Figure S3) follow for the occurrence model: three chains were run with 8000 iterations each (2000 discarded as warmup). See main text for parameter references. The trace plots

for all model parameters indicated mixing of all chains, $\hat{R}$ values were all less than 1.009 or greater than 0.999, and effective sample sizes ranged from 562 to 2198. There were no divergent transitions. We show posterior distributions or each parameter in Figure S4.

Table S1. Convergence statistics for parameters in the occurrence model.

Parameter	Effective Sample Size	Rhat
β_0	666	1.0067
β_1	1668	1.0019
β_2	1804	1.0018
β_3	632	1.0062
β_4	2198	1.0007
σ_0	562	1.0084
σ_1	1117	1.0028
b_{01}	658	1.0058
b_{02}	649	1.0060
b_{03}	643	1.0057
b_{11}	653	1.0058
b_{12}	652	1.0055
b_{13}	662	1.0056

Convergence statistics (Table S2) and trace plots (Figure S5) follow for the persistence model: four chains were run with 6000 iterations each (2000 discarded as warmup). See main text for parameter references. The trace plots for all model parameters indicated mixing of all chains, $\hat{R}$ values were all less than 1.002 or greater than 0.999, and effective sample sizes ranged from 3142 to 15,472. There were no divergent transitions. We show posterior distributions or each parameter in Figure S6.

Table S2. Convergence statistics for parameters in the persistence model.

Parameter	Effective Sample Size	Rhat
β_0	6564	1.000097
β_1	7096	1.000074
β_2	14,769	1.00047
β_3	15,472	1.00035
β_4	3486	1.0016
β_5	3142	1.00046
β_6	3398	1.00088
β_7	5509	0.999987
σ_0	3009	1.00084
σ_1	5069	1.00038
b_{01}	7282	1.00040
b_{02}	6354	1.000014
b_{03}	6266	1.00019
b_{11}	5980	1.00044
b_{12}	6564	1.00019
b_{13}	8740	0.999982

Leave-one-out cross validation was performed to evaluate the occurrence and persistence models. For the occurrence model, it was fit leaving out each combination of site and year, and, subsequently, it was used to predict a probability for the removed data. A misclassification rate of 2.8% was observed (Figure S7). Most notably, the 2019 sites that experienced blooms were misclassified, with prediction probabilities near 0.05. Though small in an absolute sense, the predicted probabilities for 2019 were larger than those observed on years without blooms.

For the persistence model, it was fit leaving out each combination of site and year at 30 days after the maximum ratio day. The fitted model was subsequently used to predict a probability for the removed data. A misclassification rate of 3.4% was observed (Figure S8).

The results of both validation procedures are approximate because a mixed effects model was fit for the validation procedure, rather than the full Bayesian model, given the relatively long fit time for the full Bayesian model. Mixed effects parameter estimates and standard errors were compared to Bayesian model posterior predictive distributions and showed general agreement. Furthermore, the model validation results are dependent upon the limited data available under bloom conditions. Both the 2015 and 2019 blooms fit the conceptual model on which the statistical model is based, and the threshold was also developed based on the characteristics of these blooms. The models will be occurrence and persistence validated in real time each year and refit with new data. However, the threshold value for the persistence model may need to be reconsidered as future data are incorporated in the model training set. If the conceptual underpinning of the model predictors is correct, then we would anticipate predictions to improve as future data are incorporated into the model training set.

Real-Time Data Source for the Ohio River cyanoHABs Shiny app

The data presented in the shiny application are derived from several public and private sources. The flow data are publicly available on the web. Flow data are retrieved from a USGS gauge station when available and from a USACE source otherwise. Table S3 provides a comprehensive list of the sites displayed by the application and the associated USGS or USACE ID and link.

The nutrient data presented on the *Supporting Evidence* tab of the application were obtained from ORSANCO (https://www.orsanco.org/data/nutrients/, accessed on 29 December 2021). The residence time data were also obtained by combining USGS flow data with estimates of pool volume provided by USACE directly to C. Nietch.

USGS water quality data with a sufficient time range were only available at Ironton, Cannelton, and Olmstead (Table S3). ORSANCO provided static data files with water quality information at Pike Island and Meldahl through July of 2019. Markland and Newburgh data are updated in real time from a direct query to the communication software used by ORSANCO to manage automatic data upload from the sensors at Markland and Newburgh.

Table S3. USGS flow data are publicly available at https://waterdata.usgs.gov/nwis/uv, (accessed on 29 December 2021), and USACE flow data are available at https://water.usace.army.mil/a2w/f?p=100:1:0:, (accessed on 29 December 2021). The data can be obtained by entering the associated ID number into the query utilities provided by each organization.

Site Name	NWS Code	USGS ID	USACE ID	Data type
Emsworth	EMSP1	03085734		USGS flow
Dashields	DSHP1	03086001		USGS flow
Montgomery	MGYP1	03108500		USGS flow
New Cumberland	NCUW2	03110690		USGS flow
Pike Island	WHLW2	03111520		USGS flow and static water quality
Hannibal	HAN01	03304300		USGS flow
Willow Island	RN001		28484108	USACE flow
Parkersburg	PARW2	03151000		USGS flow
Belleville	BEVW2		28477108	USACE flow
Racine	RACW2		28482108	USACE flow
Point Pleasant	POPW2	03201500		USGS flow
RC Byrd	GALW2		28483108	USACE flow
Huntington	HNTW2	03206000		USGS flow
Ironton		03216070		USGS water quality
Greenup	GNUK2	03216600		USGS flow
Maysville	MYVK2	03238000		USGS flow
Meldahl	MEL01		28478108	Static water quality
Cincinnati	CCN01	03255000		USGS flow
Markland	MKLK2	03277200		USGS flow and ORSANCO probe with water quality
McAlpine	MLPK2	03294500		USGS flow
Cannelton	CNNI3	03303280		USGS water quality
Newburgh	NBGI3	03304300		USGS flow and ORSANCO probe with water quality
Evansville	EVVI3	03322000		USGS flow
Smithland	SMLI2	03399800		USGS flow
Olmstead		03612600		USGS water quality

Supporting Water Data Presentations

Supporting water data presentations: (1) July 2015 nutrient loads (Figure S9); (2) residence time plots of upper river sites during the time period of the 2015 bloom (Figure S10); (3) Shiny app visualization option of stacked discharge data (Figure S11); (4) screen capture of water quality site comparison display (Figure S12); (5) screen capture of water quality year comparison display (Figure S13); (6) data for microscopy-based Ohio River algae cell counts from a period covering the 2019 cyanoHAB to provide context to the phytoplankton community structure and the cyanobacteria genera dominance (Figures S14 and S15, respectively); (7) fixed station water data time series from the Greenup pool (compliments Cody Schumacher, Marshall University (Figure S16)); and (8) 2019 Markland pool, in situ phytoplankton indicator sensor measurements made during a longitudinal

idle-speed boat survey (Figure S17). (9) nutrient concentration distributions for ORSANCO’s nutrient grab sampling program (Figure S18).

In Figure S12, the water quality data visualization options are customized to focus on the 2019 bloom season to compare dissolved oxygen and pH data at three sites for which data were available during the cyanoHAB that occurred that year (11 September to 24 October). The 2019 bloom affected the Ironton site but not the Cannelton or Olmsted sites, which are further downstream from Ironton. The variables selected of those available are known to be responsive to phytoplankton dynamics. For dissolved oxygen, when phytoplankton biomass concentrates into bloom conditions, high rates of photosynthesis during the day often supersaturates water column oxygen concentrations, while community respiration of the high biomass at night can dramatically decrease the concentrations. Therefore, when large differences between diel minimums and maximums are observed in a waterbody, excess phytoplankton is suspected. The same is true for pH, which can also be responsive to phytoplankton photosynthetic activity and be indicative of high algal biomass. We programmed a visualization of the time series of diel differences as part of the site comparison display. The graph of differences is situated under the raw variables time series plot. For the data visualization configured for Figure S12, we observe indications of more intense phytoplankton activity at Ironton compared to the other two sites.

In the screen capture shown in Figure S13, the Markland site has been selected. Markland was affected by both the 2015 and 2019 blooms. However, the time series of dissolved oxygen and diel differences are not notably different compared to other non-bloom years. This could be a function of where the oxygen sensor is installed at the Markland L&D relative to where the bulk of the bloom biomass resided. This lack of an apparent response across bloom and non-bloom years also exemplifies why water quality data may not be as directly supportive of risk probability predictive modeling as one might imagine, given a greater potential for smaller scale factors to affect the measurements compared to a more macro-scale measurement such as flow.
