# Supplementary figures and images for: Development of a Risk Characterization Tool for Harmful Cyanobacteria Blooms on the Ohio River
Source: Water (Basel). Author manuscript; Available in PMC 2022 Apr 20. (PMC9019831; doi:10.3390/w14040644)

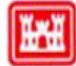

US Army Corps  
of Engineers  
Great Lakes & Ohio River Division

## Ohio River Mainstem Locks and Dams

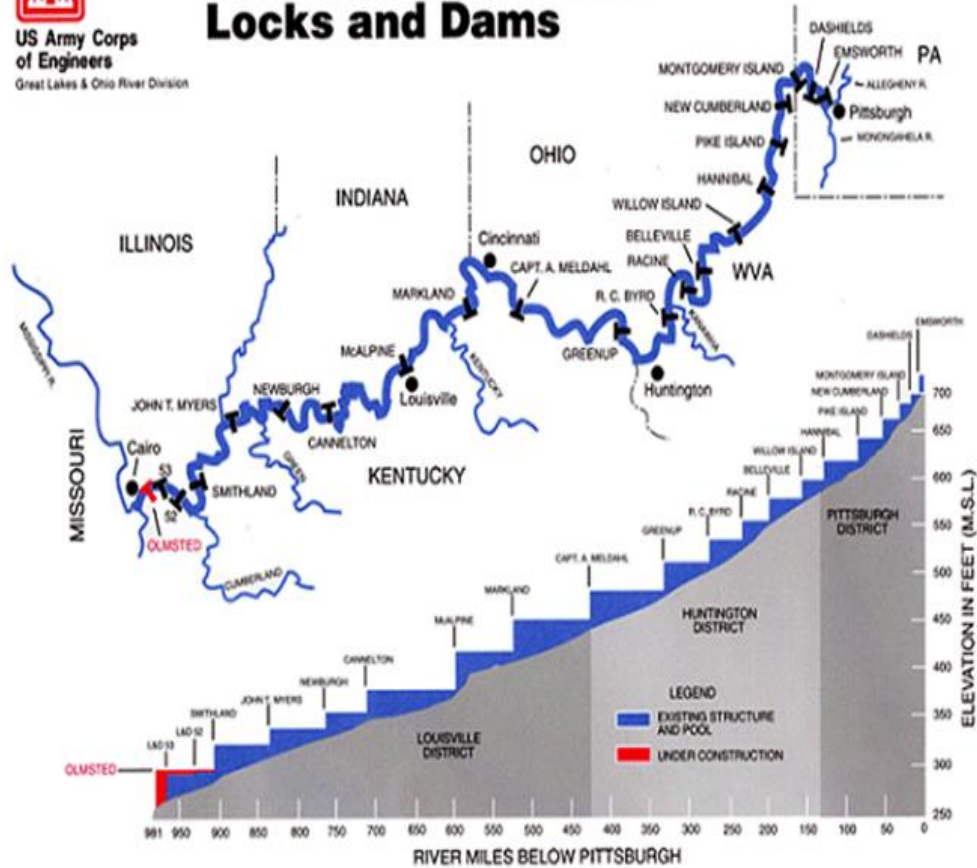

Supplement: Supplementary File [file NIHMS1783469-supplement-Supplementary_File.zip › water-1558146-supplementary-proof done/FigureS1.pdf]

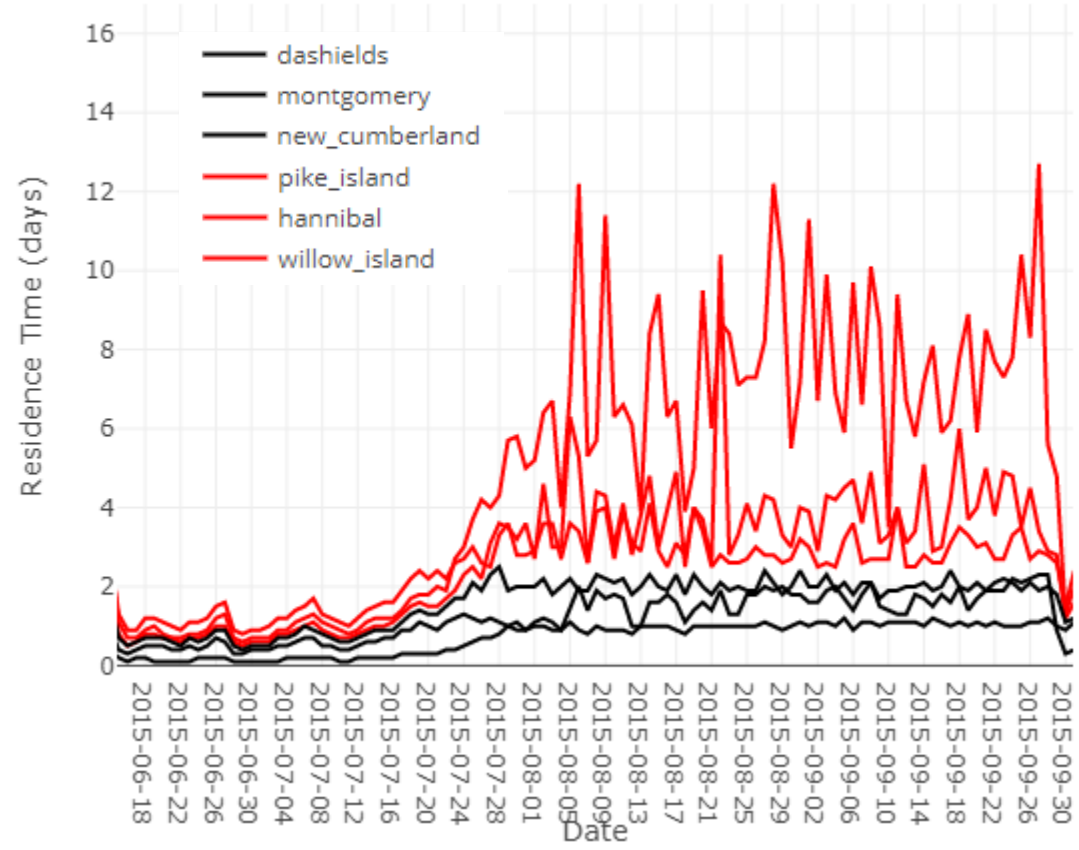

Supplement: Supplementary File [file NIHMS1783469-supplement-Supplementary_File.zip › water-1558146-supplementary-proof done/FigureS10.pdf]

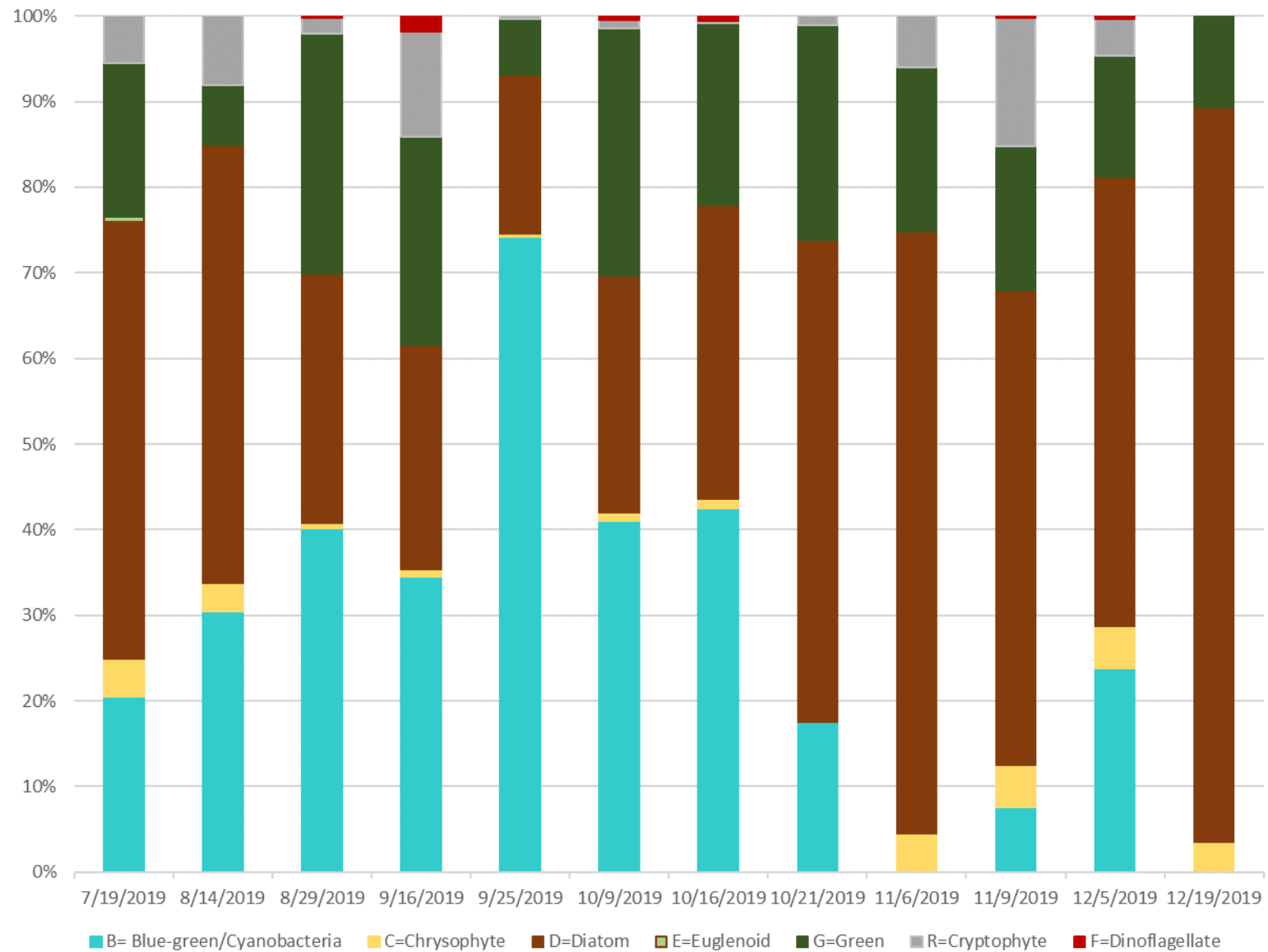

Supplement: Supplementary File [file NIHMS1783469-supplement-Supplementary_File.zip › water-1558146-supplementary-proof done/FigureS14.pdf]

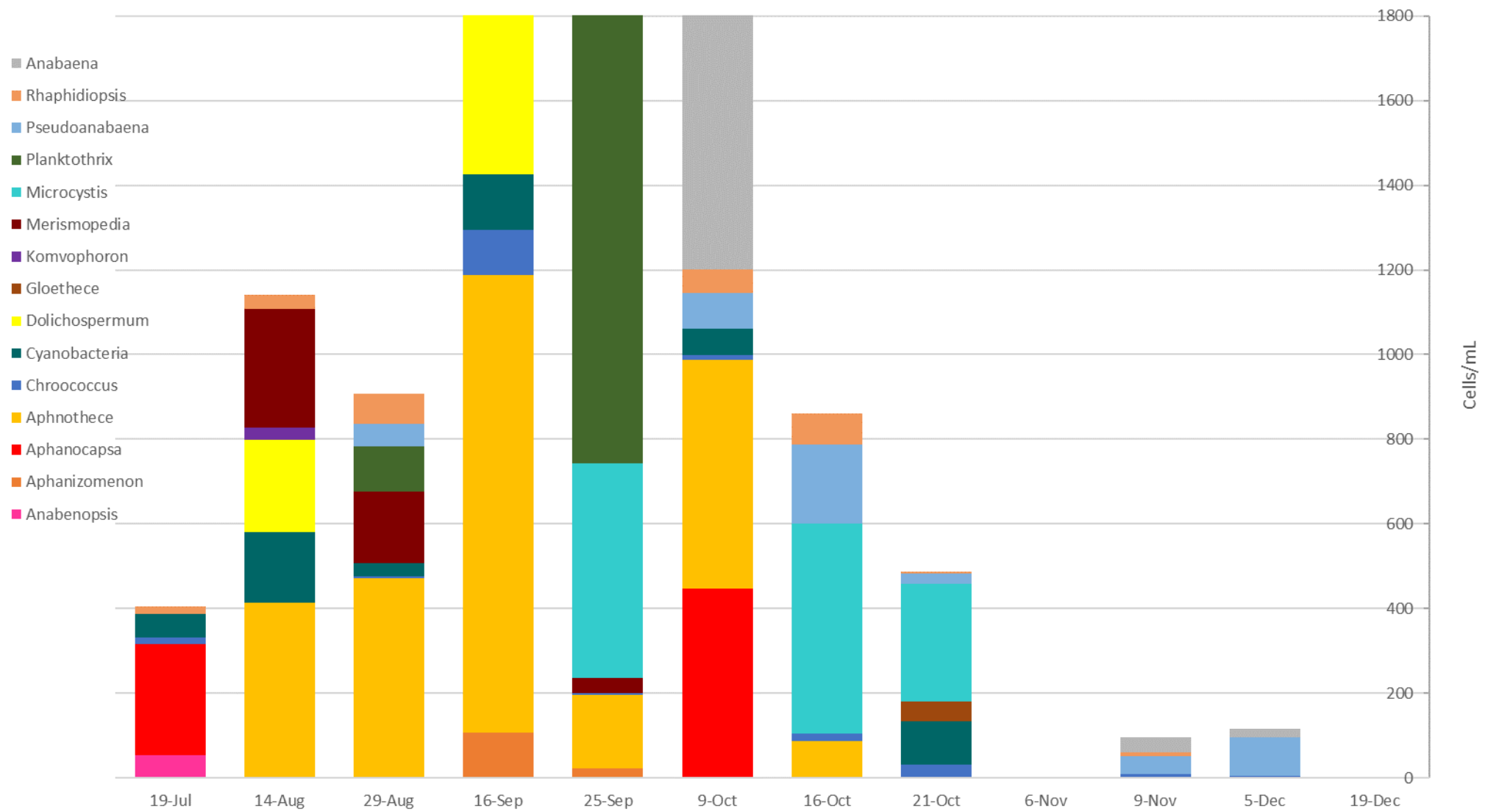

Supplement: Supplementary File [file NIHMS1783469-supplement-Supplementary_File.zip › water-1558146-supplementary-proof done/FigureS15.pdf]

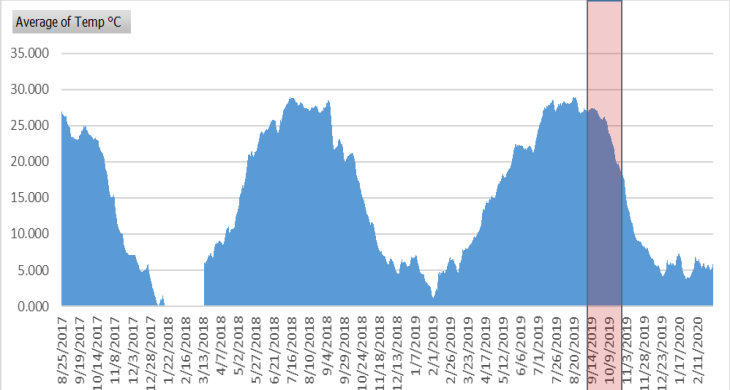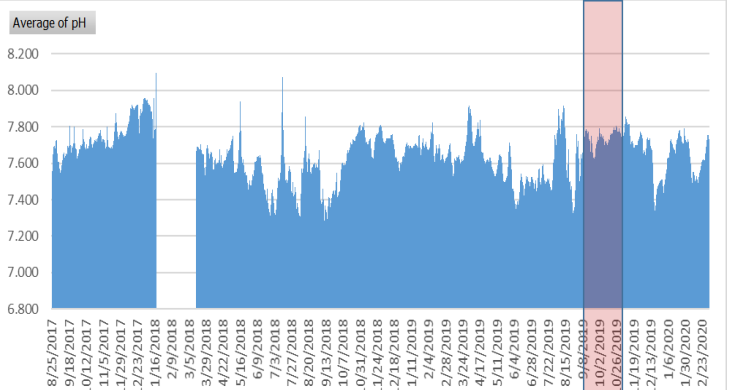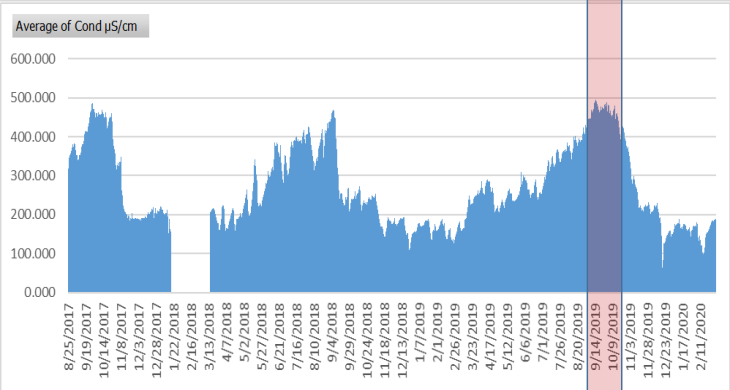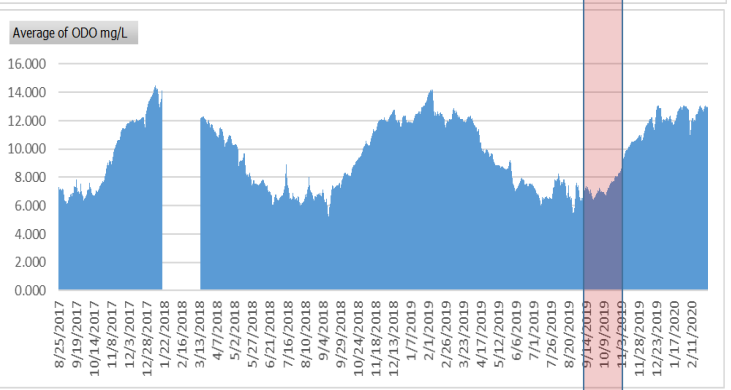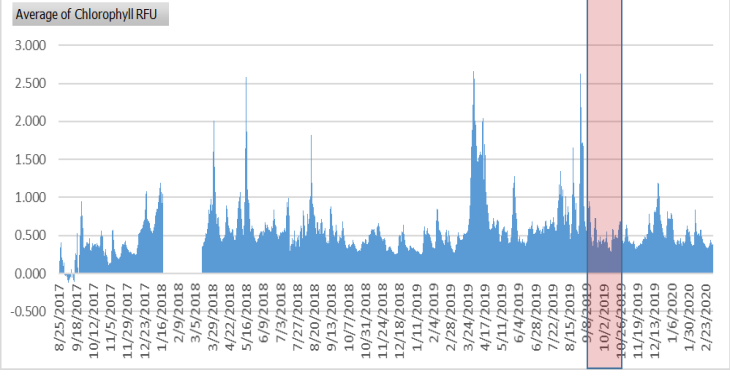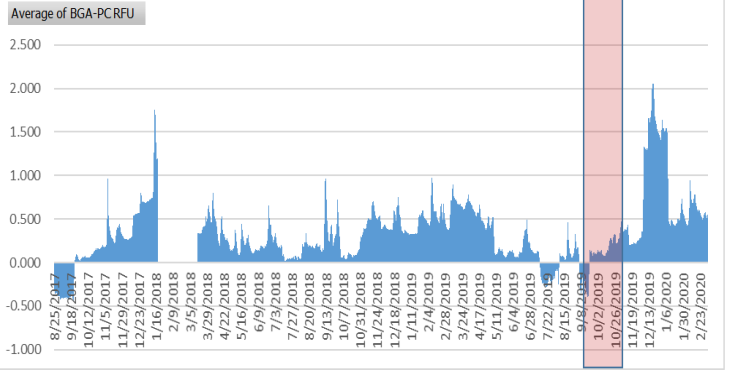

Supplement: Supplementary File [file NIHMS1783469-supplement-Supplementary_File.zip › water-1558146-supplementary-proof done/FigureS16.pdf]

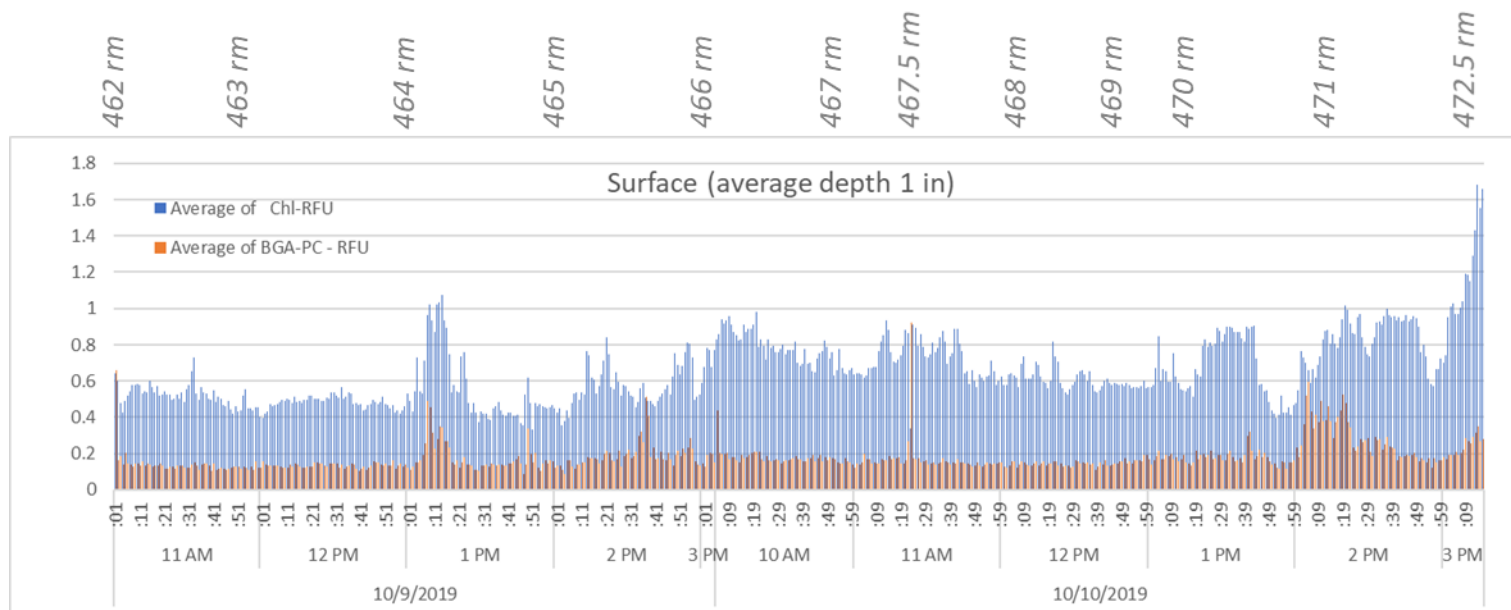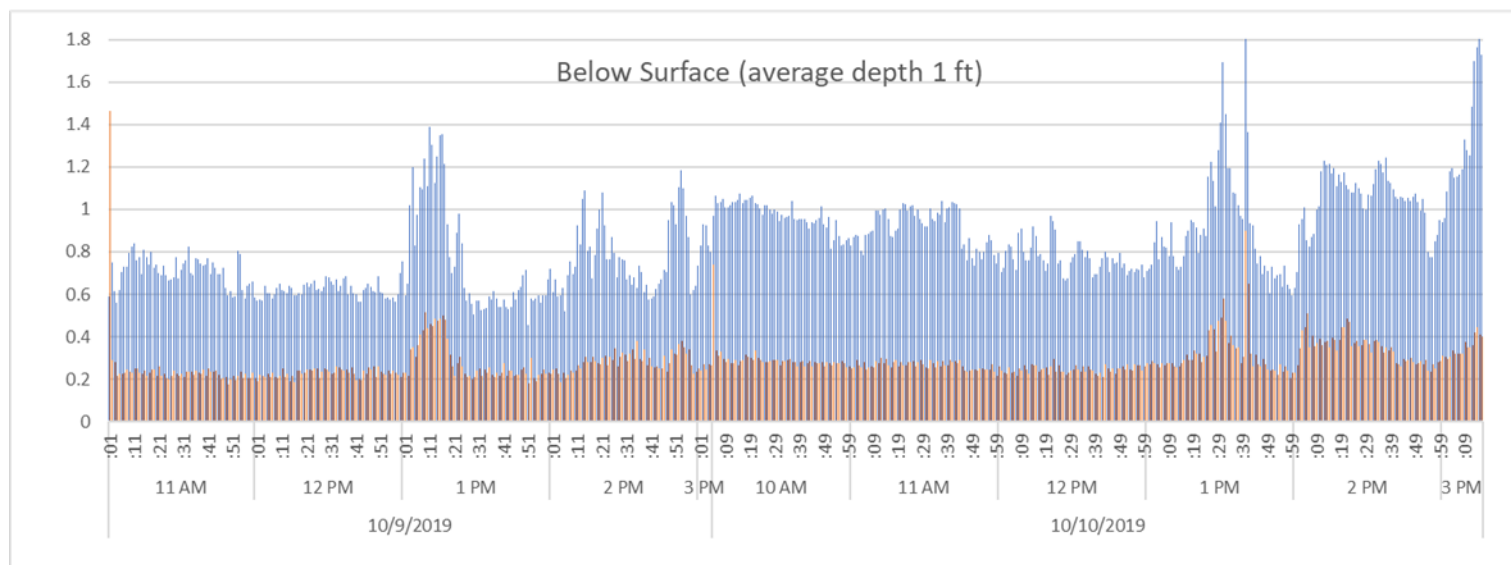

Supplement: Supplementary File [file NIHMS1783469-supplement-Supplementary_File.zip › water-1558146-supplementary-proof done/FigureS17.pdf]

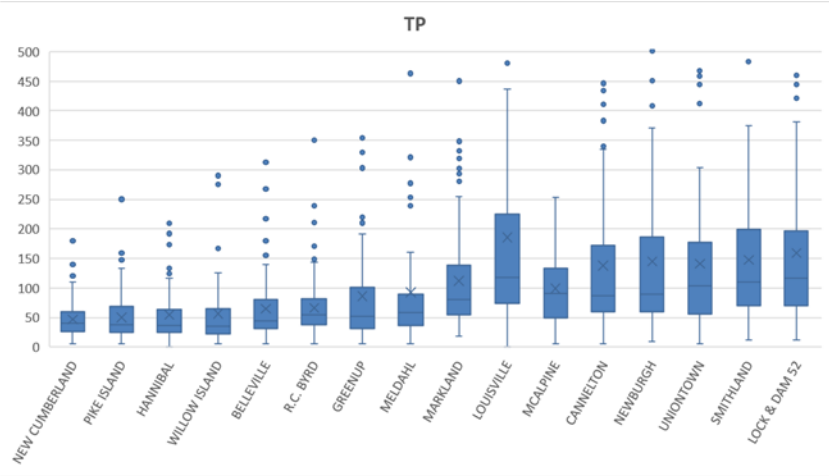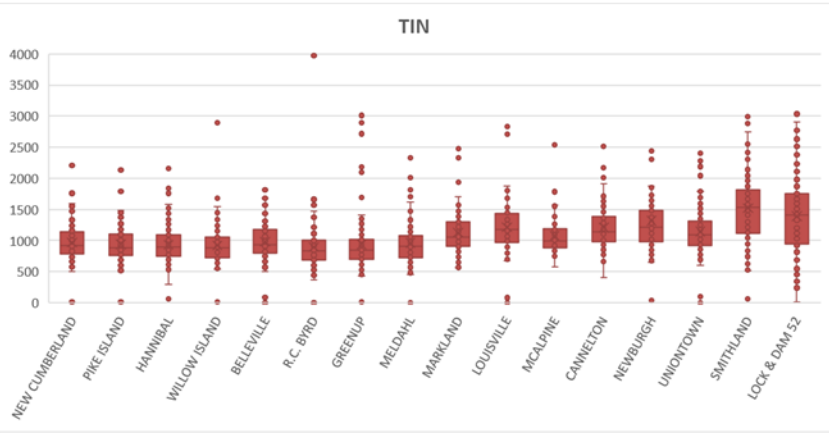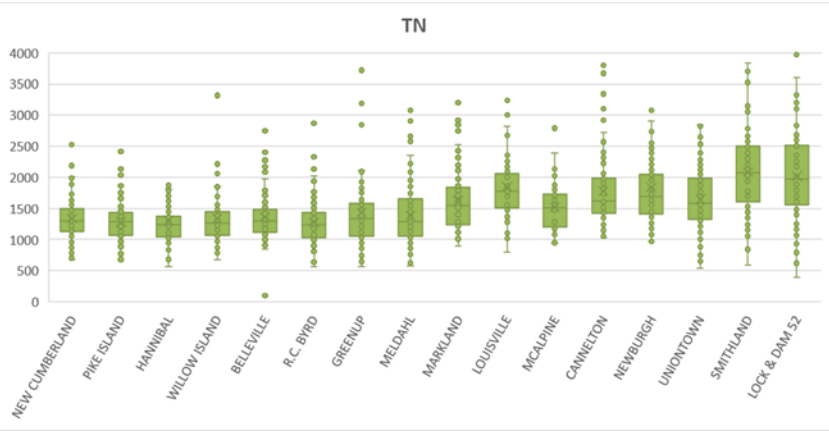

Supplement: Supplementary File [file NIHMS1783469-supplement-Supplementary_File.zip › water-1558146-supplementary-proof done/FigureS18.pdf]

(a) Meldahl L&D Upper Pool

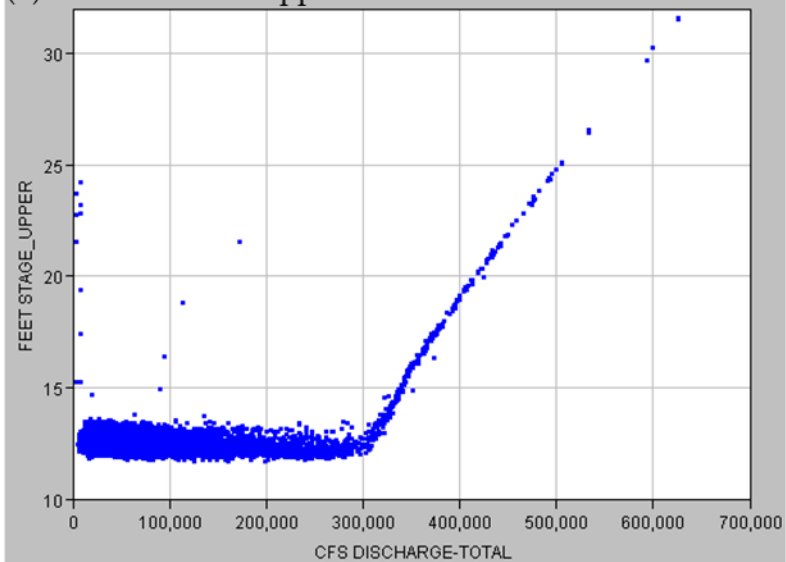

(b) Meldahl L&D Tailwater

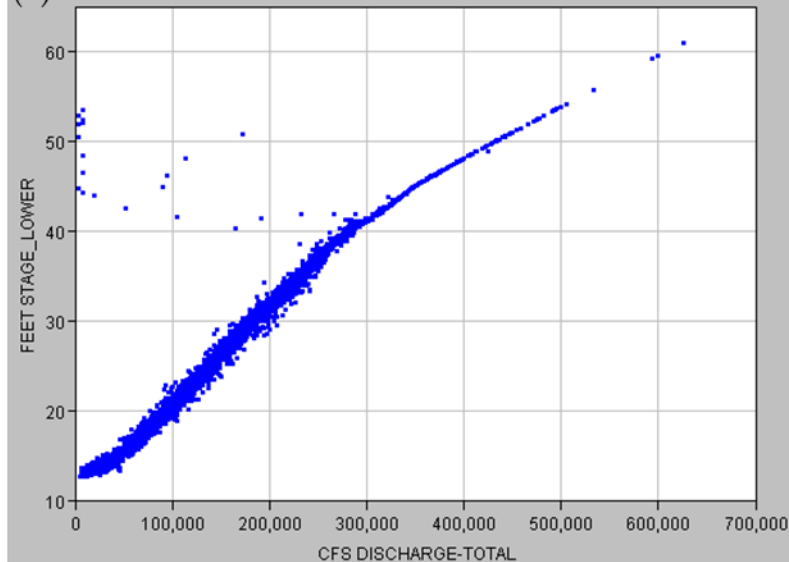

(c) Cincinnati

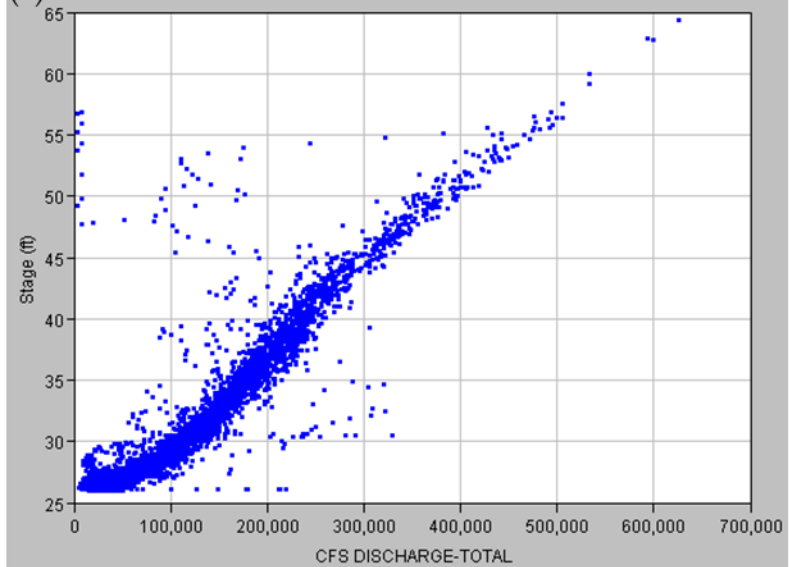

Supplement: Supplementary File [file NIHMS1783469-supplement-Supplementary_File.zip › water-1558146-supplementary-proof done/FigureS2.pdf]

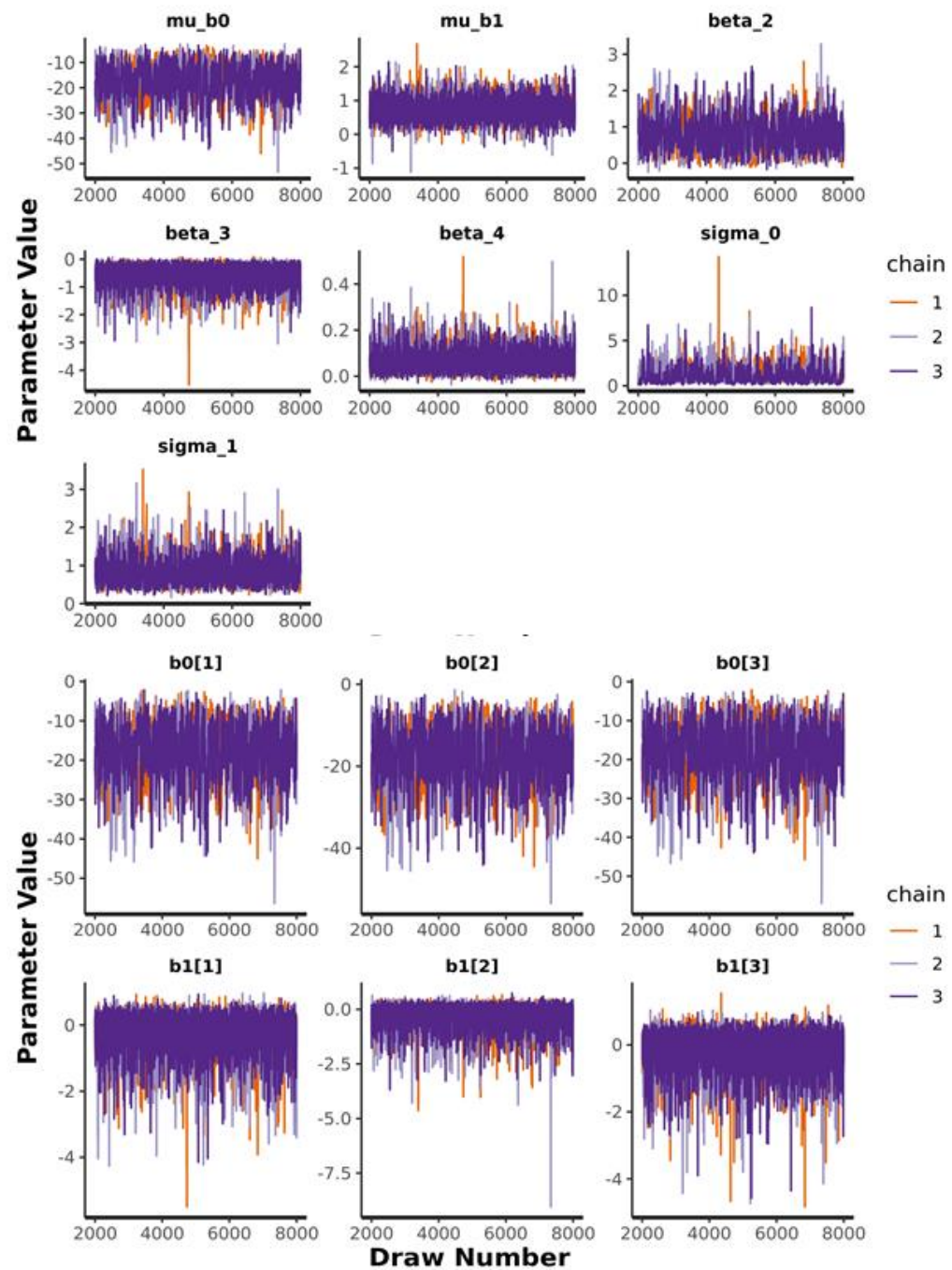

Supplement: Supplementary File [file NIHMS1783469-supplement-Supplementary_File.zip › water-1558146-supplementary-proof done/FigureS3.pdf]

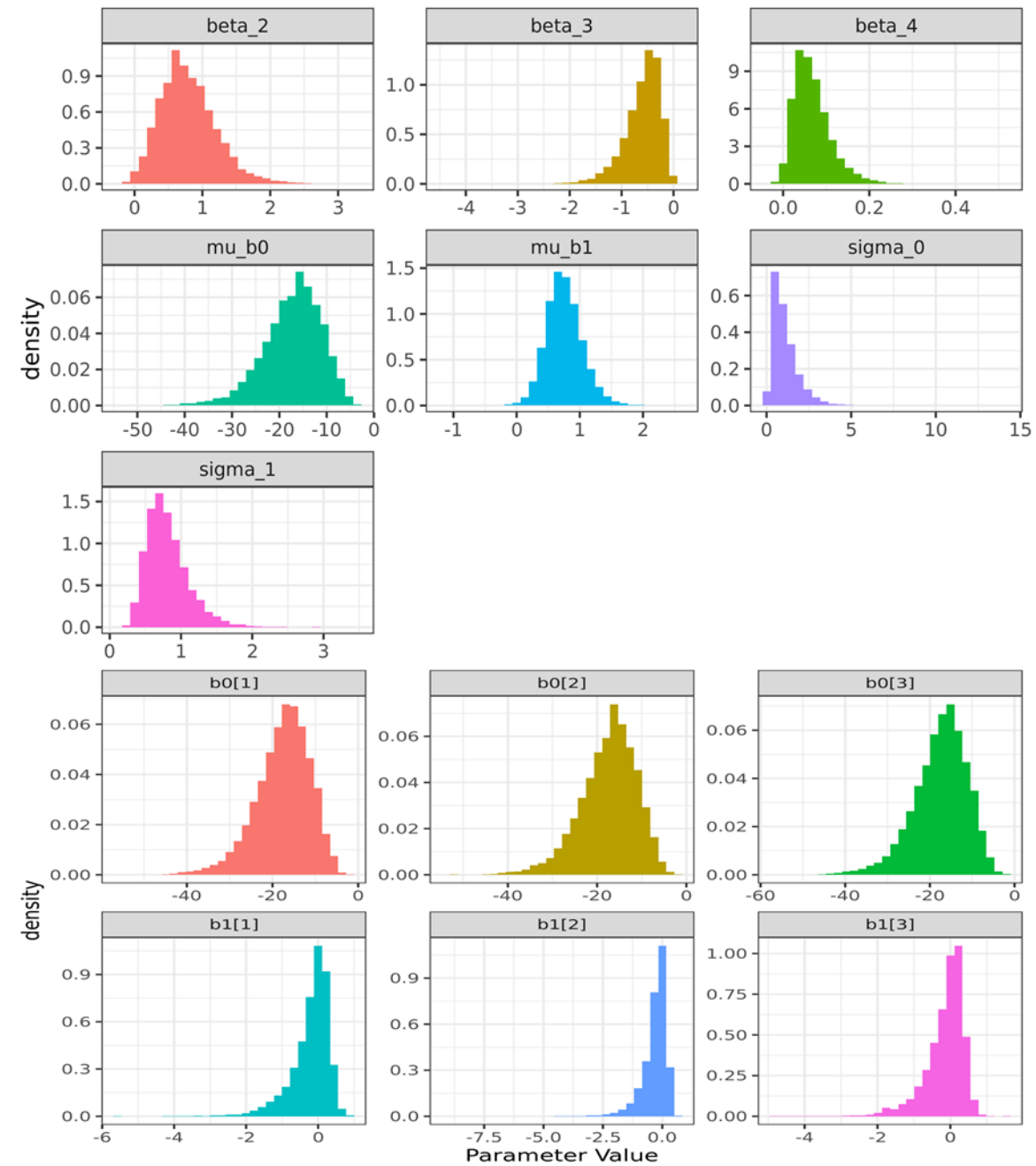

Supplement: Supplementary File [file NIHMS1783469-supplement-Supplementary_File.zip › water-1558146-supplementary-proof done/FigureS4.pdf]

Parameter Value

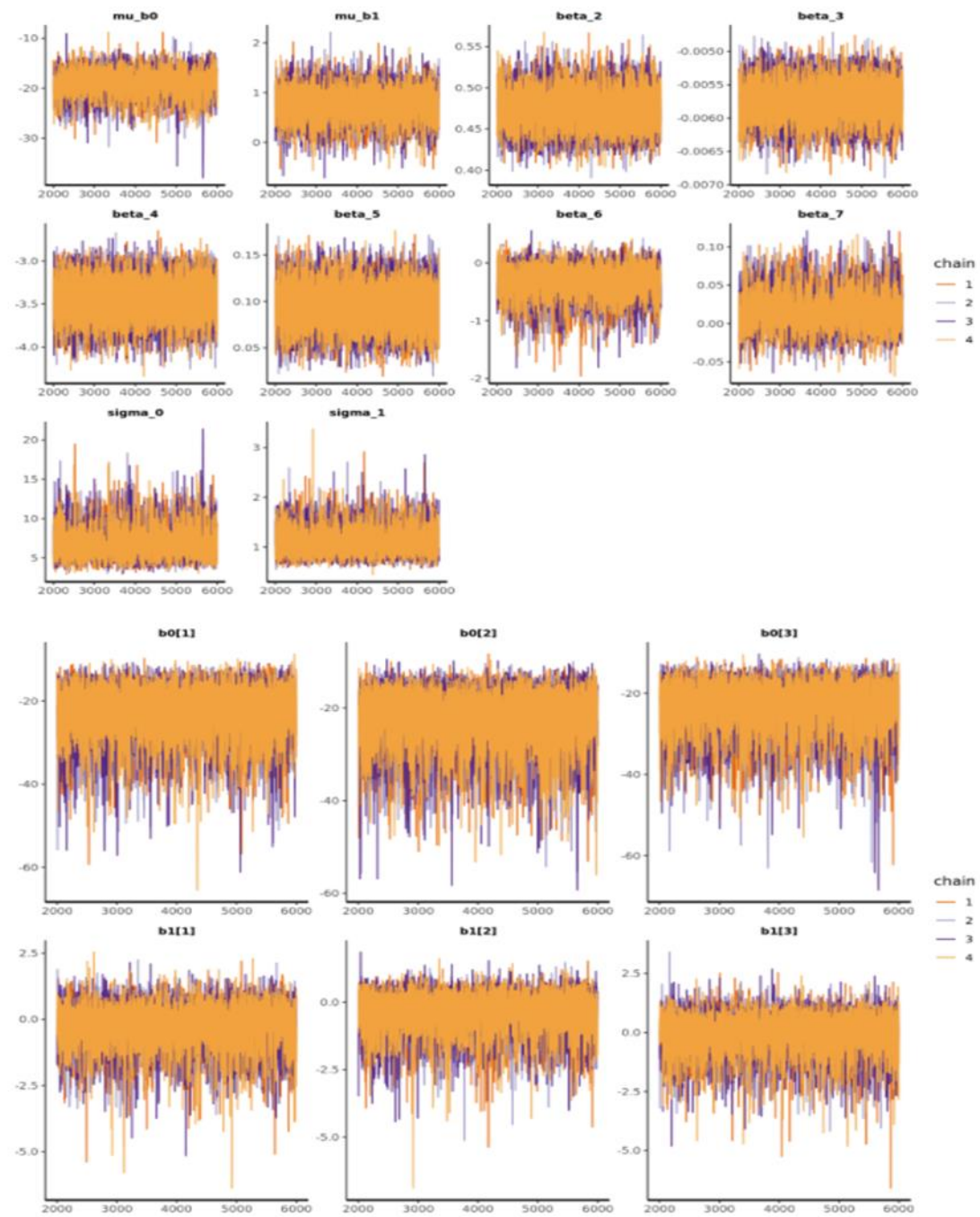

Draw Number

Supplement: Supplementary File [file NIHMS1783469-supplement-Supplementary_File.zip › water-1558146-supplementary-proof done/FigureS5.pdf]

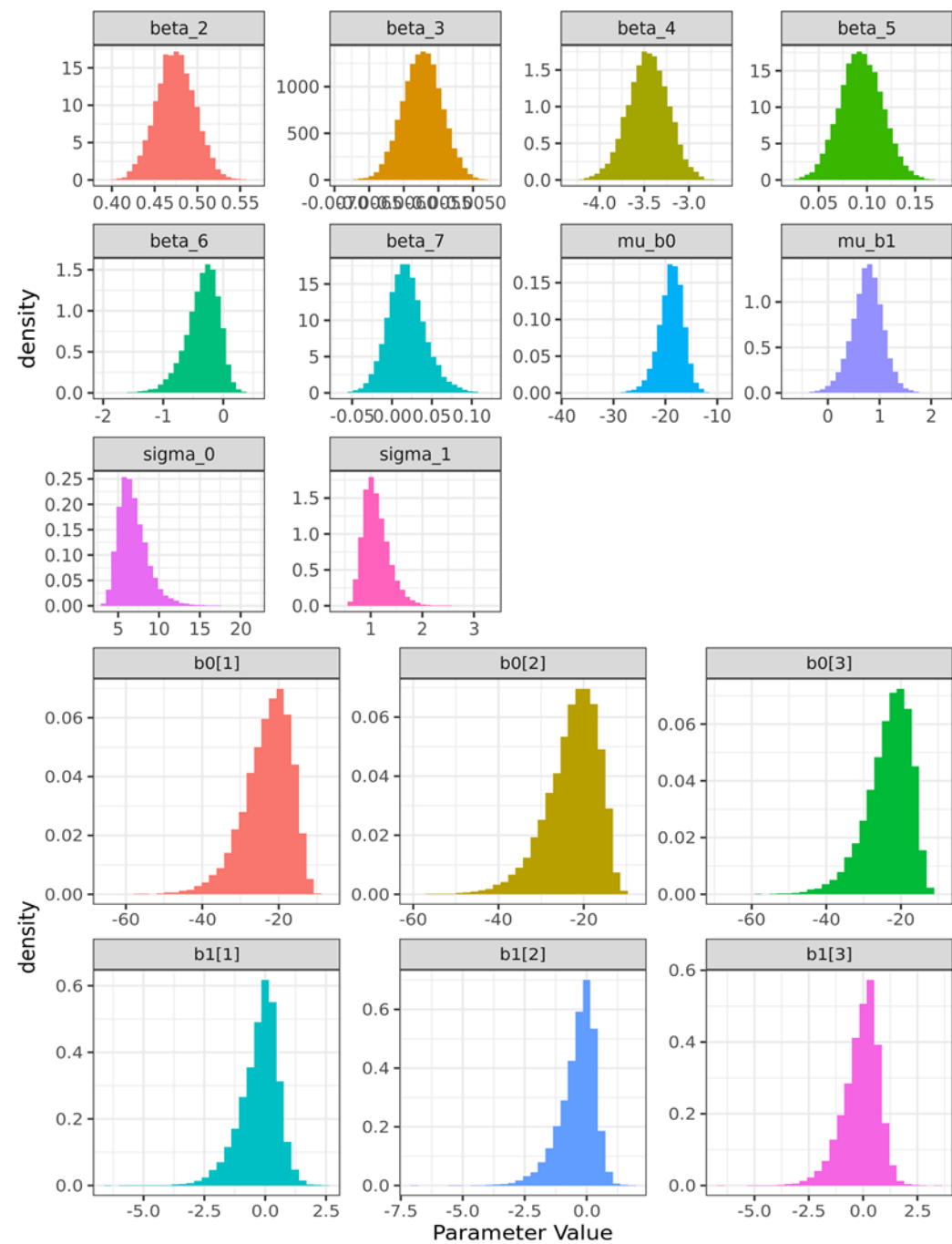

Supplement: Supplementary File [file NIHMS1783469-supplement-Supplementary_File.zip › water-1558146-supplementary-proof done/FigureS6.pdf]

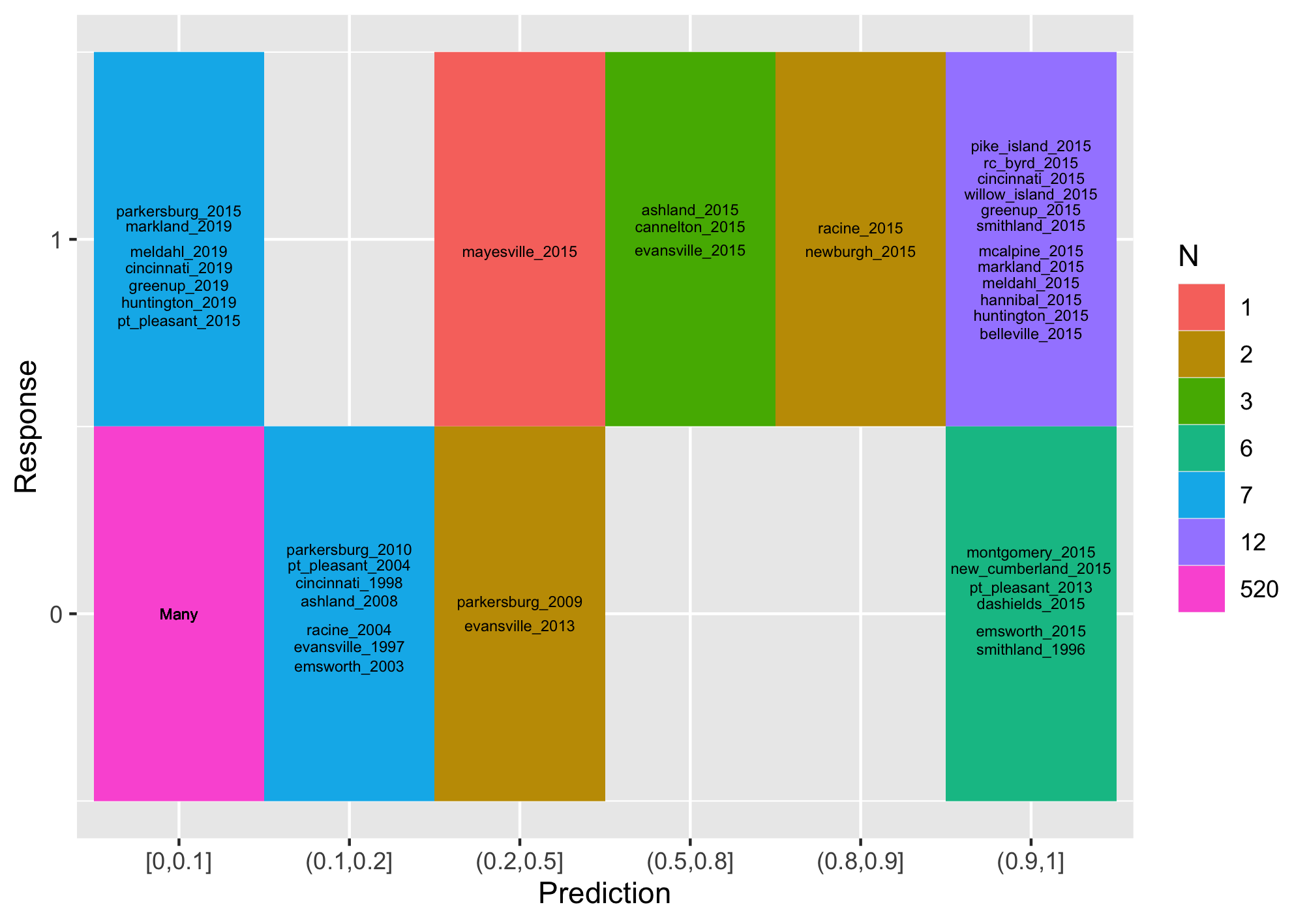

Supplement: Supplementary File [file NIHMS1783469-supplement-Supplementary_File.zip › water-1558146-supplementary-proof done/FigureS7.jpeg]

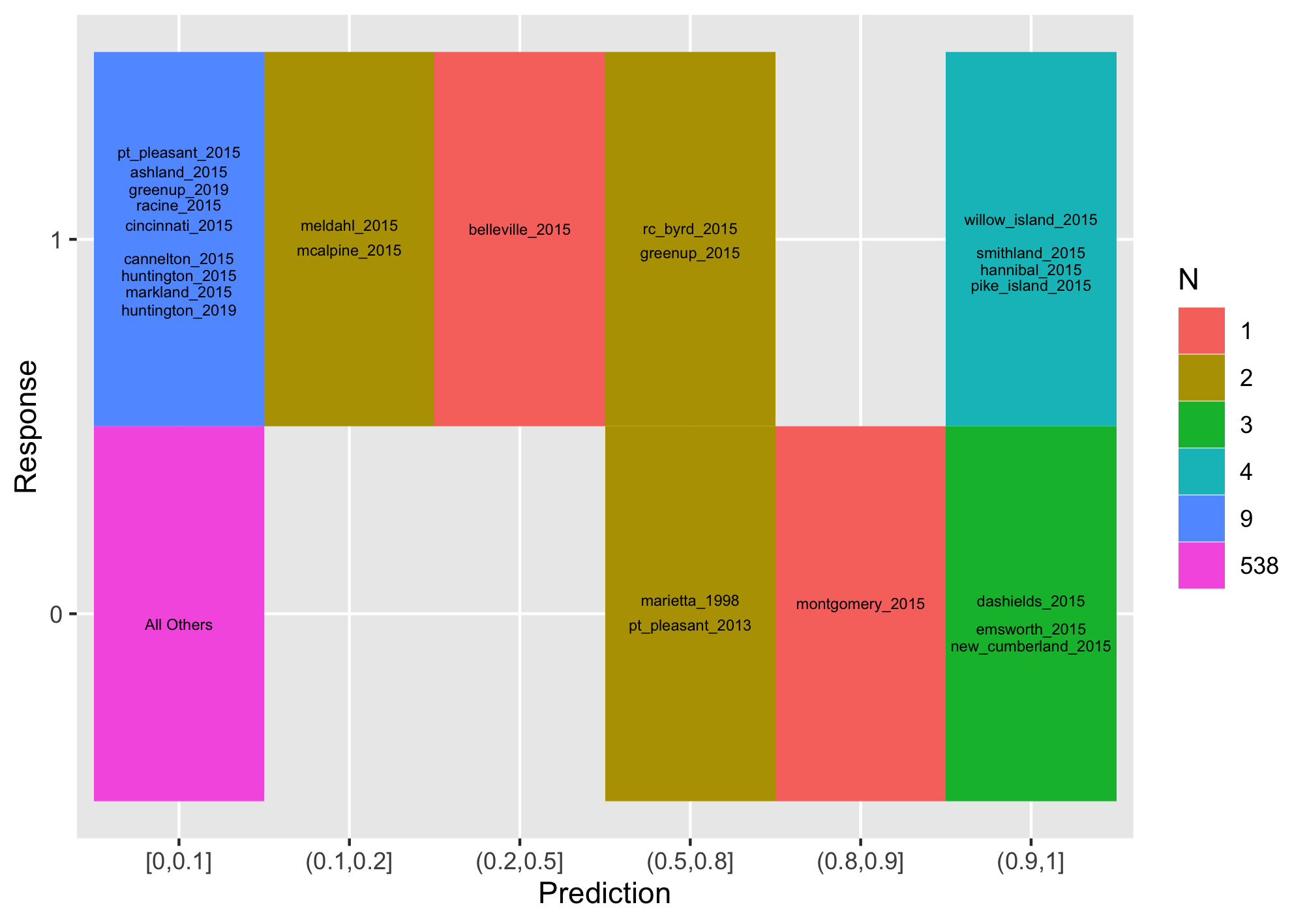

Supplement: Supplementary File [file NIHMS1783469-supplement-Supplementary_File.zip › water-1558146-supplementary-proof done/FigureS8.jpeg]

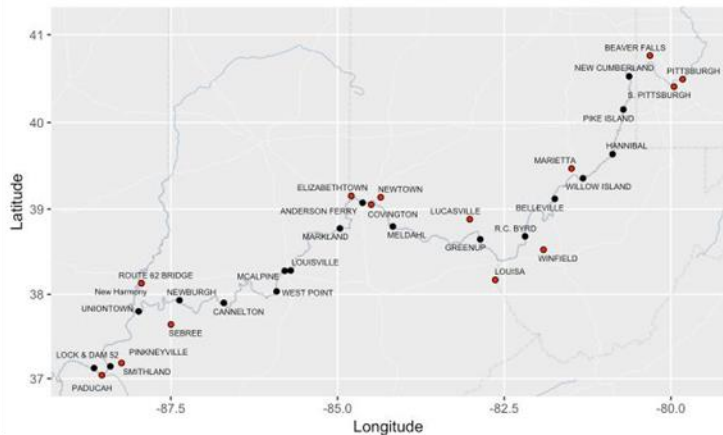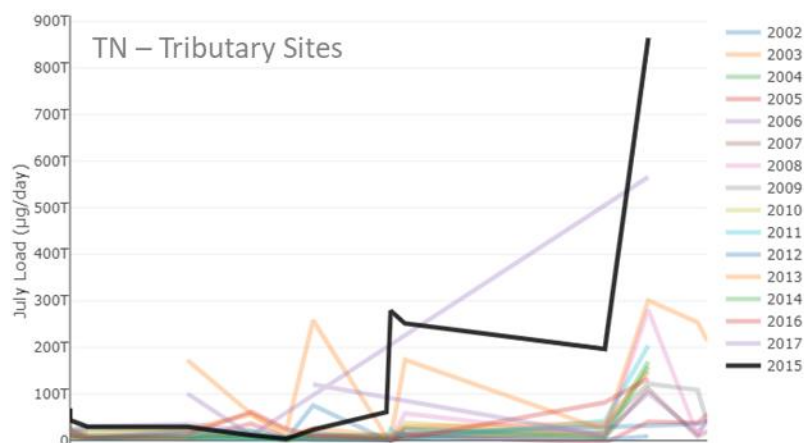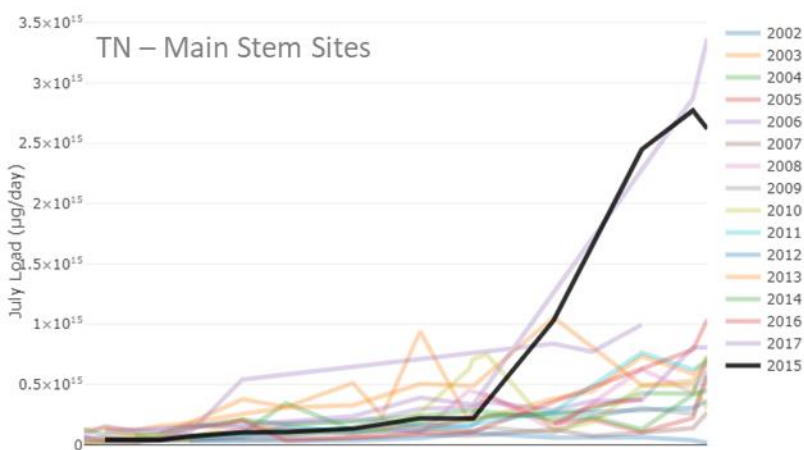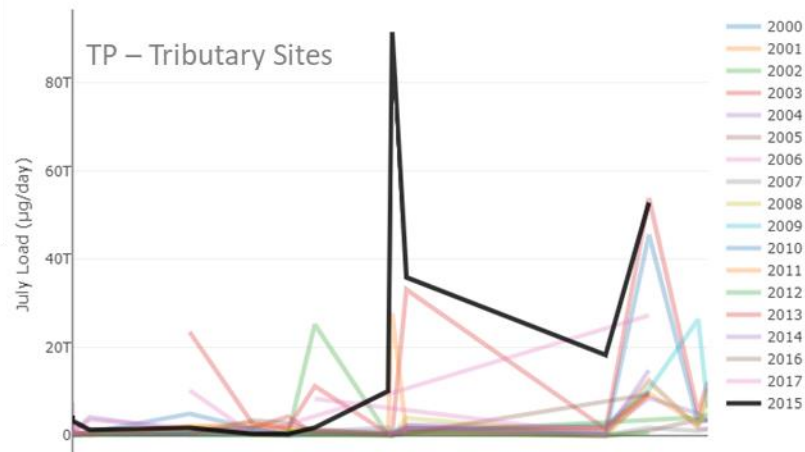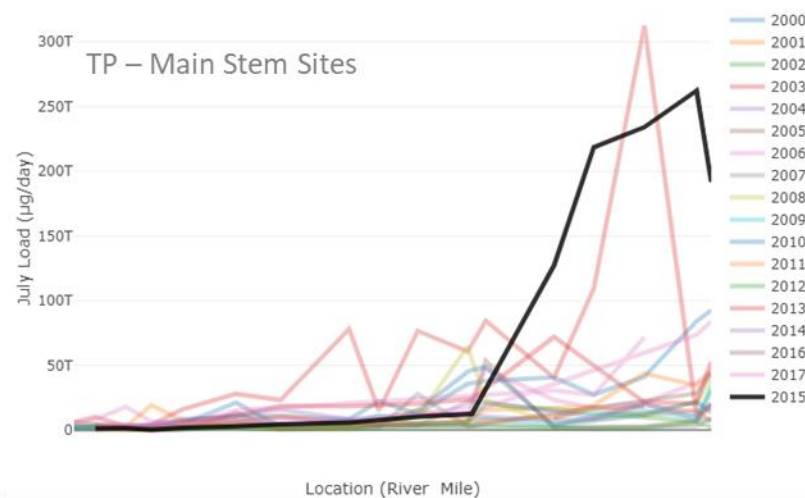

Supplement: Supplementary File [file NIHMS1783469-supplement-Supplementary_File.zip › water-1558146-supplementary-proof done/FigureS9.pdf]
